# 3D-printed intelligent photothermal conversion Nb_2_C MXene composite scaffolds facilitate the regulation of angiogenesis-osteogenesis coupling for vascularized bone regeneration

**DOI:** 10.1016/j.mtbio.2025.101647

**Published:** 2025-03-08

**Authors:** Yi Zhang, Mucong Li, Hao Zhang, Jiaqian You, Jing Zhou, Sicong Ren, Jian Feng, Yuzhu Han, Yidi Zhang, Yanmin Zhou

**Affiliations:** a Hospital of Stomatology, Jilin University, Changchun, 130021, Jilin, China; b Jilin Provincial Key Laboratory of Tooth Development and Bone Remodeling, Hospital of Stomatology, Jilin University, Changchun, 130021, Jilin, China; c Affiliated Maternal and Child Health Care Hospital of Nantong University, Nantong, 226000, Jiangsu, China; d Department of Stomatology, People's Hospital of Xizang Autonomous Region, Xizang, 850000, China; e Hospital of Stomatology, Guanghua School of Stomatology, Sun Yat-sen University and Guangdong Provincial Key Laboratory of Stomatology, Guangzhou, 510055, Guangdong, China

**Keywords:** Angiogenesis-osteogenesis coupling, Bone tissue engineering, Vascularization, 3D printing technology, Photothermal therapy

## Abstract

Personalized porous scaffold materials for bone defect repair, with adjustable mechanical strength and porosity via 3D printing technology, have made significant strides in the bone tissue engineering. However, their ability to regulate the angiogenesis processes at the defect site remains constrained, hindering the effective coupling of angiogenesis-bone regeneration. In this study, we incorporated Nb_2_C MXene as a photothermal agent and enhancer for both angiogenesis and osteogenesis, embedded into a poly (lactic-co-glycolic acid)/β-tricalcium phosphate (PLGA/β-TCP) composite biological ink. Nb releasing and precisely gentle thermotherapy successfully enhanced both angiogenesis and bone regeneration while promoting their coupling. The in vitro experiments demonstrate that the scaffold induces the upregulation of MMP family members, particularly MMP-1, MMP-3, and MMP-10, during the initial stage of bone defect repair under mild hyperthermia conditions. It promotes vascular basement membrane degradation, effectively initiating angiogenesis. Moreover, it directly activates the HIF-1/STAT3/VEGF pathway in HUVECs and triggers HSP90 expression, which stabilizes and activates the PI3K-AKT pathway in BMSCs. Consequently, this sequential linkage between PI3K-AKT and HIF-1 pathways enhances bone formation while facilitating angiogenic bone regeneration, as evidenced by the increased expression of specialized H-type vessels in rat cranial critical defect models. In vivo experimental findings further validate the effective promotion of angiogenic bone regeneration by this precision-designed PTMN scaffold under mild hyperthermia conditions, making it an effective solution for large-area bone defect repair. In summary, the precise design and manufacture of the PTMN scaffold using mild hyperthermia to fix large bone defects is a promising approach that has huge implications.

## Graphical abstract

**Figure:**
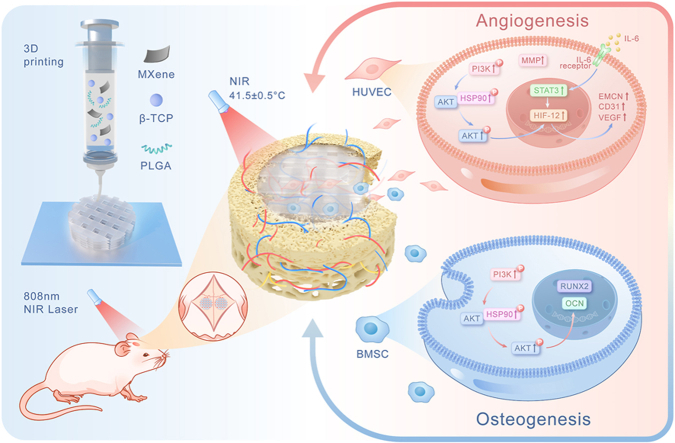

## 1 Introduction

Large bone defects arising from inflammation, trauma, and tumors have persistently posed formidable challenges in clinical practice [[1], [2], [3]]. While autologous bone grafting remains the gold standard for bone defect repair, its clinical applicability is constrained by several limitations, incongruent shape matching, requirement for an additional surgical site, and associated complications including infection, pain, nerve injury, suboptimal tissue healing at both donor and recipient sites [[4], [5], [6]]. Diverse scaffold materials for bone tissue engineering offer a minimally invasive and highly versatile alternative for repairing extensive bone defects [7,8]. Among these, 3D-printed scaffolds stand out a particularly promising solution. These scaffolds not only provide stable spatial support to the defect site, enabling the adhesion, proliferation, differentiation, and growth of bone cells, but also modulate cellular behaviors through chemical modification or drug loading and biologically active molecules [[9], [10], [11]]. By precisely matching the scaffold morphology to the defect site and optimizing the bioink composition, it is feasible to augment both the mechanical robustness and porosity of the scaffold, thereby rendering it more akin to native bone tissue [[12], [13], [14]]. This personalized bionic design has garnered escalating scholarly attention. An important question still remains, though: how can 3D-printed scaffolds help rebuild a functional vascular network within the defect site? Delayed angiogenesis impedes nutrient and metabolic waste transport, which can be solely reliant on the porous structure of the scaffold for the diffusion of these critical substances [[15], [16], [17], [18]]. An expanding body of research underscores the pivotal role of the vascular system in bone tissue regeneration, not only by facilitating the transport of essential metabolites but also by promoting the coupling of angiogenesis-osteogenesis through intricate and precise signaling transmission between endothelial cells and osteoblasts [[19], [20], [21], [22]]. This process effectively facilitates the repair of bone defects. For instance, the active formation of vascular networks promotes the enhanced secretion of the growth factor Noggin, which enhances cell recruitment and stimulates the proliferation and osteogenic differentiation of bone progenitor cells [23,24]. Consequently, one of the crucial challenges encountered by 3D-printed bone scaffolds lies in the effective reconstruction of a comprehensive and efficacious vascular network during the initial stages of bone healing.

The regeneration of the vascular system can be broadly categorized into three stages: (1) the degradation of the vascular basement membrane, mediated by metalloproteinases, which releases endothelial cells; (2) the migration, proliferation, and angiogenic differentiation of endothelial cells, culminating in the formation of novel blood vessel branches; and (3) the formation of a lumen, unimpeded blood flow, and the maturation of nascent blood vessels, ultimately culminating in the development of a functional vascular network [25]. This implies that angiogenesis is a multifaceted process, precisely regulated both spatially and temporally. At each stage, potential targets are identified to facilitate the reconstruction of the vascular network during bone defect repair. In recent years, it has become increasingly clear that endothelial cells exhibit remarkable heterogeneity and possess the ability to differentiate into various types of blood vessel. Among these, H-type blood vessels - distinguished by their elevated expression of platelets, CD31 and EMCN markers - are central to mediating the intricate interplay between angiogenesis and osteogenesis. The expression levels of these vessels are closely associated with the rate of bone healing [[26], [27], [28]]. To effectively fix extensive bone defects, it is imperative to develop a meticulously designed vascular regeneration protocol that not only promotes the expression of H-type vessels but also fosters the coupling of angiogenesis-osteogenesis.

As a novel class of 2D nanomaterials, MXenes are composed of a diverse array of carbides, nitrides, or carbonitrides. These materials have found extensive applications in the field of biomedicine, particularly in the treatment of bone tumors [29]. However, the application of MXene in bone regeneration remains relatively limited. In this study, the advantages of Nb_2_C MXene for bone regeneration are attributed to its excellent biocompatibility and biodegradability [30]. Additionally, it exhibits high photothermal conversion efficiency and stability, along with the ability to promote vascular regeneration. The biodegradation of Nb_2_C MXene is facilitated by myeloperoxidase [31], gradually releasing niobium-based substances during its degradation process. These compounds are characterized by their lower cellular toxicity, as metal elements [32], and have been shown to induce the upregulation of VEGF expression in vitro [33]. Such statement significantly enhance angiogenesis and neovascularization, as well as facilitate osteogenesis in the affected region within the organism [34]. It has been used for osteosarcoma treatment [29,35]. At the same time, Yang et al. demonstrated that when Mxene is integrated with photothermal therapy, low doses of nitric oxide (NO) at concentrations of 10^−^^9^ can be released at specific stages [35], through the physiological effects of cyclic guanosine 3′, 5′-monophosphate (cGMP) and extracellular signaling, the proliferation and migration of endothelial cells are generally regulated, thereby facilitating vascular remodeling and angiogenesis [36,37]. Nb2C MXene possesses impressive photothermal conversion performance, and remarkable photothermal stability [30]. Compared to commonly utilized photothermal conversion agents, it exhibits a larger surface area and enhanced absorbance in the near-infrared region [38]. Lin et al. evaluated the photothermal conversion efficiency of Nb_2_C MXene, reporting values 36.5 % of 1064 nm and 46.65 % at 808 nm, respectively, significantly higher than those of representative Au nanorods (21 %), Cu_9_S_5_ nanocrystals (25.7 %) [39], gold nanovesicles (37 %), and Prussian Blue (41.4 %) [40]. Additionally, as a photothermal conversion agent operating at a low temperature range of 40–42 °C, Nb_2_C MXene effectively enhances osteogenic differentiation and mineralization matrix formation of mesenchymal stem cells (MSCs). This process is intricately linked to the upregulation of heat shock proteins (HSPs) [41]. When exposed to precise temperatures, the body produces a group of proteins known as heat shock proteins (HSPs), which actively participate in cellular damage repair and enable rapid adaptation to stressful environments [42,43]. The 90 kDa heat shock protein (HSP90) is a pivotal molecule, binding to Akt and forming the Akt-HSP90 complex, which enhances the stability of Akt kinase activity and facilitates Akt phosphorylation. Consequently, activation of the PI3K/Akt pathway occurs in osteoblasts, promoting bone formation and mineralization [44]. Concurrently, the PI3K-Akt pathway stabilizes the expression of HIF-1α, further facilitating the secretion of angiogenesis-related factors like VEGF, thus establishing a connection between angiogenesis and osteogenesis.

To achieve accurate and efficient thermotherapy, while maximizing the effectiveness of vascularized bone regeneration, PLGA/β-TCP serves as the fundamental bio-ink. The PLGA scaffold exhibits excellent biocompatibility and a controllable degradation rate, which aligns with clinical requirements for a low substitution rate in bone graft materials [45]. β-TCP acts as a neutralizing agent; it can mitigate local inflammation that may arise from the reduction of microenvironment pH due to scaffold degradation, while simultaneously enhancing the mechanical properties of the scaffold and supplying calcium (Ca) and phosphorus (P) elements essential for the osteogenic process [46]. We incorporated Nb_2_C MXene as both a photothermal agent and an osteogenic promoter into the PLGA/β-TCP biological ink. Then, the customized bone tissue engineering composite scaffold, PLGA/β-TCP/Nb_2_C MXene (PTM), with superior mechanical properties, a unique porous structure, and a balanced capability for both bone conduction and induction, was fabricated using low-temperature 3D printing technology. The PTMN (PTM + NIR, PTMN) scaffold demonstrated remarkable in vitro angiogenic activity under 808 nm near-infrared radiation, promoting and initiating the reconstruction of the vascular network by upregulating MMP expression. It induced angiogenesis through direct activation of the HIF-1 pathway, stabilizing HIF-1α expression and enhancing the secretion of downstream angiogenic such as VEGF, thereby ensuring an adequate supply of oxygen and nutrient to the bone defect site. Moreover, the photothermal conversion property of Nb_2_C MXene was harnessed to recruit bone progenitor cells via controlled mild heat stimulation (41–42 °C) at the bone defect site. Activation of the PI3K/AKT/HSP90 signaling pathway enhances the synthesis of downstream bone-related proteins, including RUNX2 and COL-1, thereby augmenting the osteogenic potential of the bone progenitor cells. Moreover, the upregulation of HSP90 expression in endothelial cells further amplifies the expression of HIF-1α and VEGF, establishing a positive feedback loop that simultaneously promotes both angiogenesis and bone regeneration. In vivo experiments demonstrate that the PTMN scaffold effectively promotes the formation of CD31+EMCN + specialized H-type vessels in rats with severe cranial bone defects, facilitating coupling between angiogenesis and osteogenesis and providing a straightforward yet efficacious treatment for extensive bone defects.

## 2 Experimental methods

### 2.1 MXene characterization

The Nb_2_C MXene dispersion solution was purchased and characterized. The Nb_2_C MXene dispersion solution was diluted with ethanol solution at a volume ratio of 1:100–1:1000. After ultrasonication for 10 min, the solution was allowed to settle naturally for 5 min. The upper layer solution was dropped on a copper mesh, and the morphology was observed using a transmission electron microscopy (TEM, JEM2100F, Japan).

### 2.2 Preparation of 3D printed composite scaffolds

PLGA and β-TCP were used as the basis for the scaffold. We initially determined the optimal PLGA/β-TCP ratio of 1:1 through preliminary experiments. For detailed experimental procedures, please refer to Appendix S1-S6, Table S1, and Figs. S1–S6. First, 1 g of PLGA was dissolved in 4 mL of dichloromethane, followed by the addition of 1 g of β-TCP particles, which were sonicated for 30 min in an ice water bath. Then, the Nb_2_C MXene dispersion solution (5 mg/mL) was added to the PLGA/β-TCP suspension, adjusting the MXene content to 0 %, 0.1 wt%, and 0.3 wt%. Manual stirring for 20 min was then performed, and the mixture was loaded into the printer's hopper. The printing parameters were set as follows: layer height of 0.26 mm per layer, 5 layers, and an interlayer spacing of 1 mm. The temperature of the print cylinder was set between 20°C and 24 °C to ensure smooth printing and stable long filament deposition. The printing platform was pre-cooled to 0 °C. The initial printing speed and pressure were controlled at 8 mm/s and 0.2 Mpa, respectively. During printing, the speed and pressure were adjusted to maintain stable filament extrusion. After printing, the scaffold was frozen dried for 48 h to remove water and dichloromethane, and a stable bone tissue engineering scaffold was obtained. The scaffolds containing different concentrations of MXene (0 %, 0.1 wt%, and 0.3 wt%) were denoted as PT, 0.1PTM, and 0.3PTM, respectively.

### 2.3 Characterization of 3D printed composite scaffolds

After surface gold plating of the scaffold material, the surface and cross-sectional morphology of the scaffold material were observed using a scanning electron microscope (SEM, FlexSEM 1000, Hitachi, Japan). The element distribution in the cross-section of the scaffold was analyzed using an energy dispersive spectrometer (EDS). The chemical bonds in the scaffold material were analyzed using Fourier transform infrared (FT-IR, VERTEX 80 V, Bruker, Germany) in the spectral range of 500–4000 cm^−^^1^. The porosity of the scaffold was detected using the liquid displacement method. The scaffold was immersed in a certain volume (V_1_) of anhydrous ethanol, and the volume of the scaffold completely immersed was recorded as V_2_. After removing the scaffold, the volume of ethanol at this time was recorded as V_3_. The porosity of the scaffold was calculated using Equation S(1):

$$\text{Porosity}\;\left(\%\right)=\left(V_{1}-V_{3}\right)/\left(V_{2}-V_{3}\right)\times 100\%$$

### 2.4 Mechanical and degradation performance of scaffolds

Mechanical Performance: The compressive strength of the scaffold was tested using a universal testing machine (SHIMADZU AG-XPLUS10KN, JAPAN) with a capacity of 100 N. The scaffold was prepared into a 10 × 10 × 10 mm cylindrical shape. The compression speed was 1 mm/min, and the compression distance was stopped when 2 mm remained. The stress-strain curve was drawn to observe the compressive strength of the scaffold, and the modulus of elasticity was the initial slope of the stress-strain curve.

Degradation Performance: The initial weight of the scaffold (W_0_) was measured, and the scaffold was immersed in 5 mL of sterile PBS solution containing type II collagenase solution (1 U/mL) containing collagenase type A (1 U/mL - Roche Holding AG, Basel, Switzerland) at 37 °C in an incubator for 8 weeks. The weight of the scaffold was measured and recorded (W_t_) after freeze-drying and weighing each week. The degradation performance of the stent is evaluated using Equation:

$$\text{Residual}\;\text{mass}\;\text{ratio}\;\left(\%\right)=W_{t}/W_{0}\;x\;100\%$$

where W_t_ is the weight of the stent after degradation and W_0_ is the initial weight of the stent.

The pH value of the degradation medium of each test tube was measured with a pH meter (S20-K, USA) before and at 1-week intervals during degradation with the initial pH value of 7.4.

### 2.5 The in vitro photothermal properties of MXene composite scaffolds

To detect the photothermal performance of the scaffolds, the composite scaffolds containing different mass fractions of Nb_2_C MXene (PT, 0.1PTM and 0.3PTM) were placed in 300 μL of PBS solution. The thermal images were captured in real-time using an infrared thermal imaging camera (Shenzhen Liou Optoelectronics Technology Co., Ltd., China) under NIR (808 nm, 1.5 w.cm^−2^) irradiation and the temperature changes were recorded. To detect the thermal stability of the PTM scaffold material, the PTM scaffold material was irradiated with NIR (808 nm, 1.5 w cm^−2^) for 3 min and then naturally cooled to room temperature, and the process was repeated 5 times. The temperature changes were monitored using an infrared thermal imaging camera.

### 2.6 Cell isolation and culture

BMSCs were extracted from 7 to 10 days old SD rats (purchased from Changchun Yishi Laboratory Animal Co., Ltd. in China) and cultured. The rats were disinfected after death, and the skin and muscle were gently peeled off. The femoral, tibial, and humeral bones of the pups were obtained, and immersed in α-MEM culture medium. The epiphyseal ends were removed with an ophthalmic scissors, and the marrow cavity was flushed with α-MEM complete culture medium (10 % FBS, 1 % antibiotics/antimycotics) until it turned white. The marrow cavity flush was centrifuged (1000 rpm, 5 min), then suspended in complete culture medium and incubated at 37 °C, 5 % CO_2_ incubator. The culture medium was changed every 3 days, and the cells were passaged when the cell density reached 80 % or more. The cells were cultured to the third generation for subsequent experiments. The osteogenic α-MEM medium consisted of 50 μg/mL L-ascorbic acid, 10 × 10^−3^ M β-glycerophosphate, 100 × 10^−9^ M dexamethasone, 10 % fetal bovine serum, and 1 % PS.

HUVECs were obtained from the National Key Laboratory of Oral Diseases in Changchun, China. The cells were cultured in DMEM culture medium containing 10 % fetal bovine serum (FBS) and 1 % penicillin/streptomycin, incubated at 37 °C in a 5 % CO_2_ incubator and passaged every 4 days.

### 2.7 Cell proliferation and cell viability detection

The proliferation of BMSCs on the scaffolds was detected using a cell counting kit-8 (CCK-8, NCM Biotech Co., Ltd, China) (n = 3). The PT, 0.1PTM, and 0.3PTM groups of scaffolds (diameter 10 mm, thickness 1.5 mm) were placed in 48-well plates, with 3 replicates in each group. BMSCs were digested and centrifuged, and then seeded onto the scaffolds at a density of 1 × 10^4^ cells/well with 300 μL of α-MEM complete culture medium per well. After incubation for 4 and 7 days, the old culture medium was replaced with fresh medium, and 10 % CCK-8 reaction solution was added to each well at a ratio of 1:10 (culture medium: CCK-8 solution). The mixture was incubated at 37 °C in the dark for 1 h, after which 100 μL of the mixture was transferred to a 96-well plate and the OD was measured using an enzyme-linked immunosorbent assay (ELISA) reader (wavelength 450 nm). Similarly, the effect of NIR irradiation on the proliferation of BMSCs on the scaffolds was detected using the same method. BMSCs were seeded onto the scaffolds and allowed to adhere. The scaffolds were then irradiated with NIR (808 nm, 1.5 w. cm^−2^) for 1 min per day for 4 and 7 days. After incubation, the OD values were measured using the same method. The cell viability of BMSCs on the scaffolds was assessed by cell viability staining. BMSCs were seeded onto the scaffolds of each group and incubated for 7 days. The ratio of Calcein-AM, PI, and detection buffer was 1:1:1000. The old culture medium was removed, and the staining solution was added to make the scaffolds fully immersed in it. The mixture was then incubated in a CO_2_ incubator in the dark for 30 min. After washing the cells with PBS three times, the live and dead cells on the scaffold were observed by confocal laser scanning microscopy (CLSM, Olympus FV3000, Japan).

### 2.8 Cell morphological observation

The sterile scaffolds were placed in 48-well plates, and BMSCs were seeded on the scaffolds (PT, 0.1 PT, 0.3 PT), and incubated in an incubator. During the culture period, the scaffolds in the light group were subjected to periodic NIR (808 nm, 1.5 w. cm^−2^) irradiation, once a day, for 120 s. After 7 days of culture, the scaffolds were removed, fixed with 4 % formaldehyde for 15 min. After fixing, the scaffolds were dehydrated with different concentrations of ethanol (30 %, 40 %, 50 %, 60 %, 70 %, 80 %, 90 %, 100 % v/v) for 15 min each. The dehydrated scaffolds were frozen and dried overnight in a freeze-dryer, and then observed by scanning electron microscopy (SEM, FlexSEM 1000, Hitachi, Japan) to observe the cell morphology on the scaffolds. The same method was used to seed cells (10 × 10^4^ cells/scaffold) on the scaffolds and culture them. After 7 days of culture, all cells were fixed with 4 % formaldehyde for 10 min, then washed with PBS three times in sequence. Next, the specimen was permeabilized with 0.2 % (v/v) Triton X-100 (Solarbio, China) for 5 min. Finally, the cytoskeleton and nucleus were stained with Rhodamine-phalloidin (Solarbio, China) and DAPI (Solarbio, China), respectively, for 1 h and 10 min, respectively. The specimen was imaged using a confocal laser scanning microscope (Olympus FV3000, Tokyo, Japan) and quantitative analysis was performed.

### 2.9 Body extraction evaluation

#### 2.9.1 In vitro osteogenic effect analysis

We used real-time quantitative PCR (qRT-PCR) to detect the expression levels of bone-related genes in the scaffolds with BMSCs. The sterile scaffold material (diameter 15 mm, thickness 1.5 mm) was placed at the bottom of a 24-well plate, and BMSCs were seeded on the scaffolds at a cell density of 3 × 10^4^/well in different groups (PT, PTN, PTM, PTMN), with 5 replicates in each group. After 24 h, the old culture medium was replaced with osteogenic induction medium. The scaffolds were fully immersed in the osteogenic induction medium and the culture medium was changed every 3 days. During the culture period, the NIR-irradiated group was subjected to periodic NIR irradiation (wavelength 808 nm, 1.5 w/cm^2^), once a day, for 120 s each time. The expression of bone-related genes was detected at days 7 and 14 after culture: the cell RNA of BMSCs was extracted with TRIzol (TAKARA, Kusatsu, Japan), and the reverse transcription was performed using the TAKARA reverse transcription reagent kit (TAKARA, Osaka, Japan) according to the instructions. The quality and concentration of RNA were calculated using the NANODROP 2000c (Thermo Fisher Scientific, Fremont, CA). Finally, the polymerase chain reaction (PCR) was performed using the PrimeScript™RT-PCR reagent kit (TAKARA, Tokyo, Japan) and the Applied Biosystems 7300 (ThermoScientific, Waltham, MA). The gene primers are listed in Table S2. GAPDH was used as an internal reference, and the data were analyzed using the 2^-ΔΔCt^ method. Each experiment was repeated three times.

Alkaline phosphatase activity detection: BMSCs were seeded on the scaffolds, and the culture medium was changed to osteogenic inducing medium after 24 h. The NIR-irradiated scaffolds were subjected to periodic NIR irradiation. The alkaline phosphatase activity of the scaffolds was determined on days 7 and 14 after osteogenic induction using an alkaline phosphatase assay kit (Beyotime, China) and a BCA protein assay kit (Beyotime, China).

Expression of bone-related proteins: BMSCs were seeded on the scaffolds, and the NIR-irradiated scaffolds were subjected to periodic NIR irradiation. After 14 days of osteogenic induction, the BMSCs on the scaffolds were immunofluorescently stained. The old culture medium in the wells was aspirated, and the scaffolds were washed with PBS three times. The scaffolds were fixed with 4 % formaldehyde for 15 min, washed, and then incubated with an immunostaining blocking solution (Beyotime, Shanghai, China) for 1 h. The blocking solution was aspirated, and the diluted primary antibodies were added: OCN rabbit monoclonal antibody; COL-1 rabbit monoclonal antibody; RUNX2 rabbit monoclonal antibody; OCN rabbit monoclonal antibody, and they were incubated at 4 °C overnight. The primary antibodies were recovered, washed with PBS three times, and Alexa Fluor 488-conjugated goat anti-rabbit IgG (1:2000; Beyotime, Shanghai, China) was added and incubated at room temperature in the dark for 1 h. The secondary antibodies were recovered, washed with PBS three times, and rhodamine-phalloidin (Solarbio, China) was added and incubated at room temperature in the dark for 1 h. Recover phantom ring peptide, wash it with PBS three times, then add DAPI (Solarbio, China) at room temperature in the dark and incubate for 5 min. After rinsing, observe the sample using CLSM and quantify the fluorescence intensity using Image J software. The experiment was repeated three times.

To further investigate the osteogenic mechanism of photothermal therapy, Western Blot analysis was used to analyze the expression of relevant proteins. BMSCs were seeded on the scaffolds of each group, and the irradiation group was irradiated with NIR (808 nm, 1.5 w cm^−2^) for 120 s per day. After 7 days of culture, the cells were digested, centrifuged, and collected. The cells were then incubated with a homogenization solution (RIPA: protease and phosphatase inhibitor mixture = 1000:1) to fully homogenize the cells, and the supernatant was obtained by centrifuging (14800 rpm, 15 min). The protein sample was obtained. The protein concentration was determined by the BCA method for subsequent experiments. The protein sample and protein loading buffer (4:1 ratio) were mixed proportionally, boiled at 100 °C for 5–10 min, and an upper layer gel and a lower layer gel were prepared in proportion. The electrophoresis buffer was added to the inner and outer wells, and the markers were added to both ends. The protein samples were added in order in the middle, with 20–30 μg of protein added to each well. The electrophoresis was started, with an initial voltage of 70 V. The voltage was increased after 30 min. The electrophoresis was stopped when bromophenol blue was near the bottom of the glass plate. The proteins were transferred from the gel to a PVDF membrane using wet transfer method, with the transfer voltage and time set at 300 mA and 90 min, respectively. After the transfer was completed, the membrane was incubated with blocking solution for 30 min at room temperature to block non-specific binding. The membrane was cut according to the molecular weight of the proteins and placed in the appropriate primary antibody (4 °C overnight incubation: β-actin, Akt, p-Akt, HSP90). The primary antibody was recovered, washed, and incubated with the secondary antibody (HRP-conjugated secondary antibody) at room temperature for 1 h. The secondary antibody was recovered, washed, and ECL substrate was added. Then observe the results through a developing machine.

#### 2.9.2 In vitro angiogenesis performance

The Transwell assay was used to evaluate the migration ability of HUVECs. HUVECs were digested with trypsin, centrifuged, and resuspended in DMEM culture medium containing 2 % FBS. 2 × 10^4^ cells/well were added to the upper chamber of a 24-well Transwell culture plate. A sterile support was placed at the bottom of the lower chamber, and DMEM culture medium containing 10 % FBS was added to cover the bottom of the upper chamber completely. The Transwell culture plate was incubated in an incubator for 12 h, and the upper chamber was fixed with 4 % paraformaldehyde for 15 min. The upper chamber was washed with PBS 3 times, dyed with 1 % crystal violet (Beyotime) for 10 min, and gently wiped with a cotton swab to remove the cells from the upper chamber membrane. The upper chamber was washed with PBS 3 times, and the number of cells and the extent of migration were observed under a microscope and photographed. Quantitative analysis of HUVECs migration was performed using the ImageJ 00.6.0 software on randomly selected 3 fields of view. The experiment was repeated 3 times.

Using Matrigel as the basal membrane matrix substrate, a tube formation experiment was conducted to determine the effect of different groups on the tube-forming ability of HUVECs. The 48-well plate and the tip of the pipette were pre-cooled in the freezer at −20 °C, and then the Matrigel was thawed at 4 °C for 24 h. The 48-well plate was placed on an ice box, and 100 μL of Matrigel was added to each well. After confirming that there were no bubbles, the plate was placed in the cell culture incubator for 0.5 h to allow the matrix gel to solidify. Cell seeding: HUVECs were seeded on the supports of each group and pre-treated for 3 days. After digesting the cells from each group, they were suspended and added to the 48-well plate at a density of 4 × 10^4^ cells/well, which had been coated with matrix gel. The plate was placed in the incubator for culture. The tube formation effects of endothelial cells on the matrix gel were observed and recorded under a microscope (400 magnification, Olympus, Japan) at 4 h and 8 h. Three random fields were selected and the number of nodes and tube length were calculated using the imagejvol 6.0 software.

We used RT-qPCR technology to detect the expression levels of angiogenesis-related genes on the stent. The sterile stent material was placed at the bottom of a 24-well plate, and HUVECs were seeded on the stent at a density of 50 × 10^4^ cells/well. The illumination group stent was subjected to periodic NIR irradiation (808 nm, 1.5 w/cm2), once a day, for 120 s. The expression of angiogenesis-related genes was detected 3 days after seeding. RNA was extracted using TRIzol (TAKARA, Kusatsu, Japan), and reverse transcription was performed using the TAKARA reverse transcription kit (TAKARA, Osaka, Japan) according to the instructions. The quality and concentration of RNA were calculated using the Thermo NANODROP 2000c (Thermo Fisher Scientific, Fremont, CA). Finally, the polymerase chain reaction (PCR) was performed using the PrimeScript™RT-PCR kit (TAKARA, Tokyo, Japan) and the Applied Biosystems 7300 (ThermoScientific, Waltham, MA) according to the instructions. β-actin was used as an internal reference, and the data were analyzed using the 2^-ΔΔCt^ method. Each experiment was repeated three times. The primer sequences are shown in Table S3 and Table S4.

Immunofluorescence staining of HIF-1α, HUVECs were separately cultured on PT, PTM, and PTMN scaffolds for 3 days, then fixed with 4 % paraformaldehyde for 15 min, washed with PBS 3 times, and blocked with immunostaining blocking solution (Beyotime, Shanghai, China) for 1 h at room temperature. Then, the specific HIF-1α rabbit monoclonal antibody (1:500; diluted with one-antibody dilution buffer) was incubated at 4 °C overnight, and Alexa Fluor 488-conjugated goat anti-rabbit IgG (1:2000; Beyotime, Shanghai, China) was incubated at room temperature for 1 h. The cell cytoskeleton and nucleus were stained with rhodamine-phalloidin (Solarbio, China) for 1 h, and DAPI (Solarbio, China) for 5 min. All staining images were captured by an inverted microscope (Olympus, Japan). The fluorescence intensity of cell immunostaining was evaluated using ImageJ software. The experiment was repeated three times.

Western blot analysis was used to detect the expression of HSP90 and HIF-1α proteins in HUVECs. The same method was used for protein blot analysis in BMSCs.

#### 2.9.3 Establishment of rat cranial bone defect model

Twenty-four SD rats (7-week-old; weight 200–250 g) were selected for the experiment and housed for 3–5 days before the animal experiment. The ethical approval number was KT20200. The rats were randomly divided into 4 groups: blank group, PT group, PTM group, and PTM + NIR (PTMN) group. The rats were weighed, and anesthetized with 10 % aqueous potassium chloride (2 mL/kg) intraperitoneally. The rats were shaved and the surgical site was sterilized. The skin was incised along the longitudinal suture, the periosteum was separated, and the skull was exposed. Using a burr, symmetrical critical-sized bone defects (diameter 5 mm) were created on the sagittal suture of the skull on both sides using the skull as a reference. The sterilized support was placed in the bone defect in the PTMN group, and no support was placed in the blank group (sham operation group). The incision was then sutured in layers. After surgery, the rats were injected with antibiotics (10,000 U/day) continuously for 3 days. The PTMN group was subjected to regular NIR (808 nm, 1.5 w/cm^2^) treatment, once every other day, for 600 s each time, with the temperature maintained at approximately 41–42 °C using a thermal imaging instrument to record temperature changes.

The rats were sacrificed at 4th and 8th weeks post-operation, and all procedures were strictly conducted in accordance with the requirements of animal ethical treatment. After the cranial bone specimens were fixed in 4 % paraformaldehyde for 48 h, they were scanned using Micro-CT (mCT-50, SCANCO MEDICAL Inc., Switzerland) and quantitatively analyzed. The parameters were set as follows: 70 kV, 200A, 300 ms integration time, 1 mm aluminum filter, and spatial resolution of 27 μm. After scanning, the samples were decalcified with 10 % EDTA for 4 weeks, embedded in paraffin, and then sectioned for H&E, Masson, immunohistochemistry, and immunofluorescence staining. To further investigate the mechanisms of angiogenesis and osteogenesis, we performed α-SMA/CD31 co-immunofluorescence staining, EMCN/CD31 co-immunofluorescence staining, OPN, and CD31 immunohistochemistry analysis on the tissue sections. Finally, images were captured using an inverted microscope (Olympus, Japan) and evaluated using Image j. The PT, PTM, and PTMN stents were implanted subcutaneously in JCR mice, and at 8 weeks, the hearts, lungs, kidneys, liver, and spleen of the mice were collected and stained with H&E to evaluate the potential toxicity of the different stents in vivo.

### 2.10 Statistical analysis

The data were expressed as mean ± standard deviation. Statistical analysis was performed using SPSS 19.0 software (SPSS, Chicago, IL). According to the number of comparisons made by GraphPad Prism Software 6, a one-way ANOVA followed by Tukey's multiple comparison test was used for statistical difference analysis. Results with statistical significance were indicated by ∗p < 0.05, ∗∗p < 0.01, ∗∗∗p < 0.001, and ∗∗∗∗p < 0.0001. All experiments were repeated three times.

## 3 Results and discussion

### 3.1 Preparation and representation of the PLGA/β-TCP/Nb_2_C MXene (PTM) composite scaffolds

**Fig. 1:**
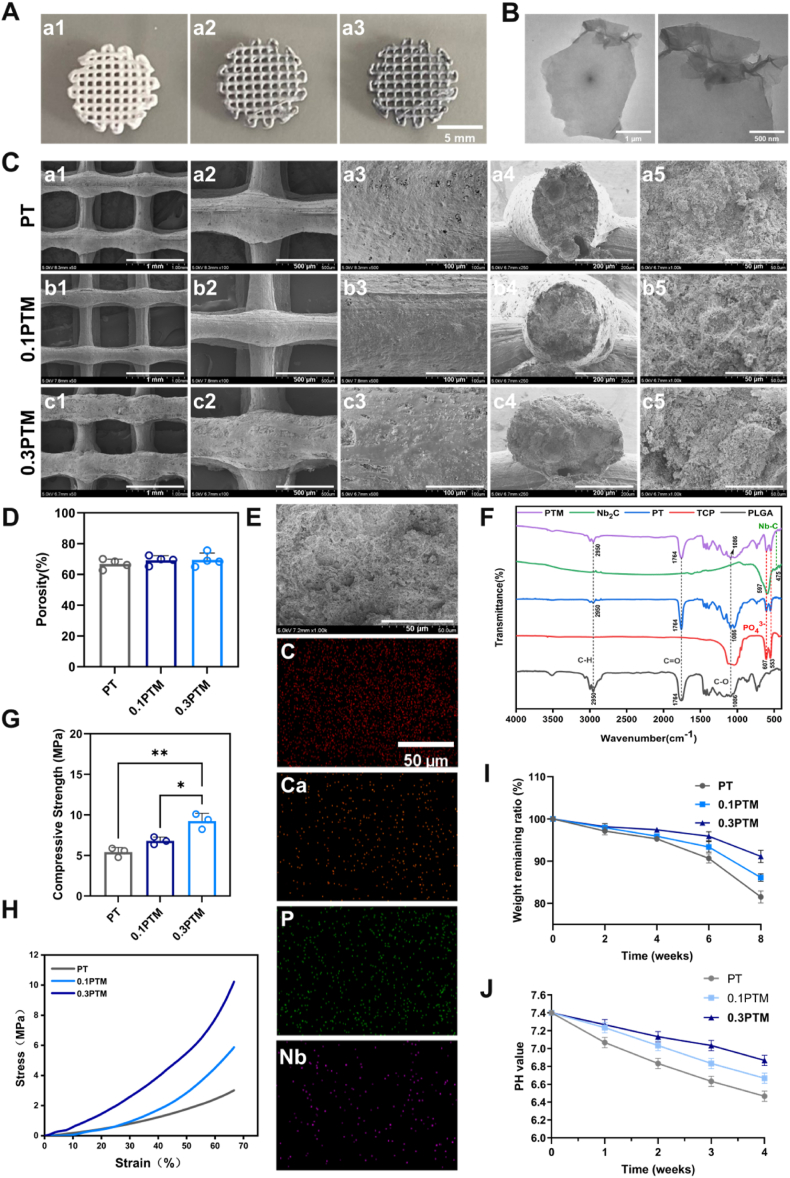
The characterization of PT, 0.1PTM, and 0.3PTM. A) Digital photographs of a1: PT; a2: 0.1PTM; a3: 0.3PTM. B) SEM image of Nb_2_C MXene nanoplate. C) SEM images of the surface (1–3) and cross-section (4–5) of the PT/0.1PTM/0.3PTM. D) Quantitative analysis for the porosity of PT, 0.1PTM, and 0.3PTM. E) The SEM image and corresponding EDS element distribution map for the 0.1PTM stent. F) FT-IR spectra are compared to show differences among β-TCP, PLGA, Nb_2_C MXene, PT, and 0.1PTM. G) quantitative analysis on compressive strength. H) quantitative analysis on compressive stress-strain curves. I) degradation rate-time curve respectively for PT, 0.1PM, 0.3PTM. J) pH value-time curve during degradation for PT, 0.1PM, 0.3PTM. (*∗p* < 0 00.05, *∗∗p* < 0.01, *∗∗∗p* < 0.001, *∗∗∗∗p* < 0.0001, “ns” indicates no significant difference, n = 3, error bars represent mean ± standard deviation).

Nb_2_C MXene (One technology Co., LTD, China) was integrated into the scaffolds as the photothermal agent and osteogenic enhancer, and PLGA/β-TCP/Nb_2_C MXene (PTM) composite scaffolds were prepared through 3D printing with the mass fraction of Nb_2_C MXene adjusted to 0 %, 0.1 wt%, and 0.3 wt%. The synthesized scaffolds were named PT, 0.1PTM, and 0.3PTM, respectively. Digital photos of the composite scaffolds can clearly exhibit the printed 3D structure (Fig. 1A). Intuitively, from PT, 0.1PTM, to 0.3PTM, the color of different scaffolds gradually changed from white to black. With Nb_2_C MXene as the photothermal agent, the 3D-printed scaffold microstructure supplies a favorable microenvironment for angiogenesis and bone induction. The efficient photothermal conversion characteristics of 2D Nb_2_C MXene in near-infrared light have been extensively proven. The transmission electron microscope (TEM) reveals that Nb_2_C MXene is a monolayer with an ultrathin 2D lamellar structure (Fig. 1B). The scanning electron microscope (SEM) was utilized to observe the surface morphology of the scaffolds, and micron-sized pores were detected on various scaffolds. The PT scaffold had a relatively smooth and dense surface, and unlike the PT scaffold, both the 0.1PTM and 0.3PTM scaffolds featured rougher surfaces (Fig. 1C a3-c3; the thumbnails are shown in Fig. S7, Supporting Information). Numerous studies have demonstrated the advantageous impact of this micro-nanostructure on promoting the osteogenic differentiation of bone marrow-derived mesenchymal stem cells [[47], [48], [49], [50]]. The cross-sectional view illustrates the good quality of the connected pores of the prepared scaffolds (Fig. 1C a4-c4). Based on the porosity test, these scaffolds had a porosity above 65 %, contributing to the growth of the blood vessels and the delivery of nutrients (Fig. 1D) [51,52]. Additionally, the porosity of the PT scaffold was 66.80 ± 2.70 %, the 0.1PTM scaffold 69.23 ± 2.53 %, and the 0.3PTM scaffold 69.43 ± 3.85 %, with values comparable to that of the natural cancellous bone [53]. However, no significant difference was found between these prepared scaffolds. The SEM image and corresponding element content mapping reported the distribution of elements in the cross sections of the 0.1PTM scaffold, demonstrating the even distribution of carbon, calcium, phosphorus, and niobium in the scaffold (Fig. 1E). This substantiates the successful loading of Nb_2_C MXene into the scaffolds. The chemical composition of the PTM composite scaffold was further illustrate through the Fourier transform infrared spectroscopy (FTIR) (Fig. 1F). Moreover, the characteristic peaks of Nb_2_C MXene appeared at 597 cm**^−1^** and 475 cm**^−1^**, representing Nb-O and Nb-C, respectively. For PLGA, the peak at 2950 cm**^−1^** represented the telescopic vibration of C-H, and those at 1086 cm**^−1^** and 1764 cm**^−1^** stood for the telescopic vibration of C=O and C-O, respectively. Then, for β-TCP, the peaks at 607 cm**^−1^** and 553 cm**^−1^** represented PO**_4_^3−^**. In this paper, PT simultaneously had the characteristic peaks of β-TCP and PLGA, while PTM had the characteristic peaks of β-TCP, PLGA, and Nb_2_C MXene, denoting the successful synthesis of the PTM composite scaffolds.

Bone tissue engineering composite scaffolds also necessitate appropriate compressive strength. We conducted a compressive strength test to identify the mechanical properties of the scaffolds. The compressive strengths of PT, the 0.1PTM and 0.3PTM scaffolds were 5.40 ± 0.41 MPa, 6.79 ± 0.37 MPa, and 9.24 ± 0.77 MPa, respectively (Fig. 1G). Meanwhile, the stress-strain curve displays that the compressive elastic modulus of the scaffold was also proportional to the content of Nb_2_C MXene (Fig. 1H). In line with the above results, Nb_2_C MXene fortified the mechanical strength of the scaffold because the abundant modifiable functional groups on Nb_2_C MXene's surface combined with PLGA/β-TCP matrix through π-π electron cloud, Van der Waal's force, and other chemical bonds, thereby stabilizing the structure and enhancing the scaffold's mechanical properties. Bone conductivity, bone induction, and bone formation are the three fundamental criteria for assessing the quality of replacement materials used in bone defect repair [54]. Bone conductivity refers to the spatial support capability of scaffold materials to guide the growth of new blood vessels and the migration of osteoblasts, thereby facilitating new bone formation. This necessitates that the scaffolds possess an appropriate degradation rate; excessively rapid degradation would result in the loss of spatial support, potentially causing bone formation disorders, whereas overly slow degradation may provide insufficient space for new bone growth and delay the bone formation process [55]. In vitro, the PT scaffold degraded at the fastest rate. The weight remaining ratio is 80.50 ± 1.15 % in the 8th week. Equally important, the degradation of the scaffold containing Nb_2_C MXene was relatively slow, particularly the 0.3PTM scaffold whose mass decreased by only 8.86 ± 1.19 % during the 8th week (Fig. 1I). We observed that during the 6th to 8th week of in vitro degradation, the degradation rate of all three groups of scaffolds increased significantly. This acceleration can be attributed to the hydrolysis of ester bonds in PLGA [56], where acidic byproducts such as lactic acid and glycolic acid generated during degradation act as catalysts for further ester bond hydrolysis, leading to a self-accelerating degradation process [45]. Additionally, we monitored the pH changes in the buffer solution throughout the degradation period (Fig. 1J). The results indicated that the presence of Nb_2_C MXene slowed the decline in pH, which explains the relatively slower degradation rates observed in the 0.1PTM and 0.3PTM scaffolds compared to PT. Studies have demonstrated that Nb_2_C MXene exhibits excellent stability under varying pH and temperature conditions, maintaining its performance within a pH range of 4–10 [57]. Consequently, the pH fluctuations resulting from scaffold degradation are unlikely to compromise the functionality of Nb_2_C MXene. Overall, after eight weeks, the degradation rate of all three scaffold groups remained below 20 %, comparable to deproteinized bovine bone mineral (DBBM), thus meeting the clinical requirements for bone graft materials with low resorption rates, pointing out that the composite scaffold was sufficiently stable to support long-term bone regeneration.

### 3.2 Photothermal properties of PTM composite scaffolds

**Fig. 2:**
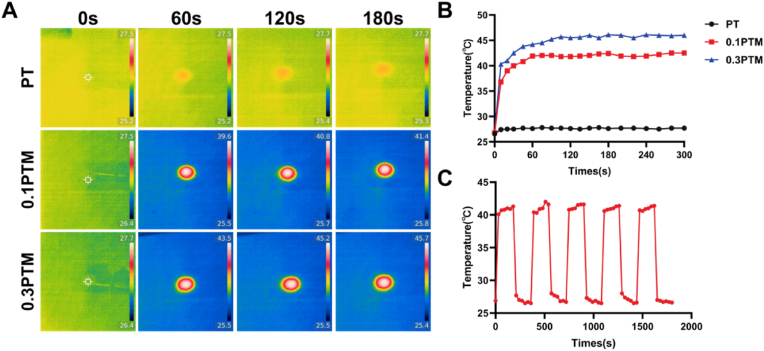
A) Real-time thermal imaging images of PT, 0.1PTM, and 0.3PTM under near-infrared radiation (1.5 w/cm^2^). B) Temperature change curves of PT, 0.1PTM, and 0.3PTM under near-infrared radiation (1.5 w/cm^2^). C) Recycling heating profile of 0.1PTM composite scaffold under 808 nm laser irradiation (1.5 w/cm^2^) for five laser on/off cycles**.**

The impact of Nb_2_C MXene on the photothermal properties of the scaffolds was investigated by employing an infrared thermal imager to monitor real-time temperature changes under near-infrared (808 nm, 1.5 w/cm^2^) illumination (Fig. 2A). The temperature of the 0.1PTM scaffold rapidly elevated to the bone-forming threshold (41.2 ± 0.8 °C) within 60 s, subsequently reaching a steady state. Under identical conditions, the temperature of the 0.3PTM scaffold swiftly rose to 45.7 °C within 60 s and then stabilized, while negligible fluctuations in temperature were observed for PT scaffold under near-infrared illumination (Fig. 2B). The findings indicate that with an increase in the content of Nb_2_C MXene in the scaffold, there is a significant and rapid rise in temperature. The incorporation of Nb_2_C MXene has endowed the composite scaffold with commendable photothermal performance. Excellent photothermal stability was also detected after conducting five cycles of laser irradiation of 0.1PTM scaffolds using 808 nm NIR light (1.5 W cm^−2^, 3 min for each cycle) (Fig. 2C). The maximum temperature of the 0.1PTM scaffold remained approximately at 41 °C, which corresponds to a mild thermal stimulation (41–42 °C) known for promoting bone regeneration [42,[58], [59], [60]]. Therefore, the PTM scaffold showcases stable and efficient photothermal conversion performance and can be effectively employed for photothermal treatment of bone defects.

### 3.3 Biocompatibility of PTM scaffolds

**Fig. 3:**
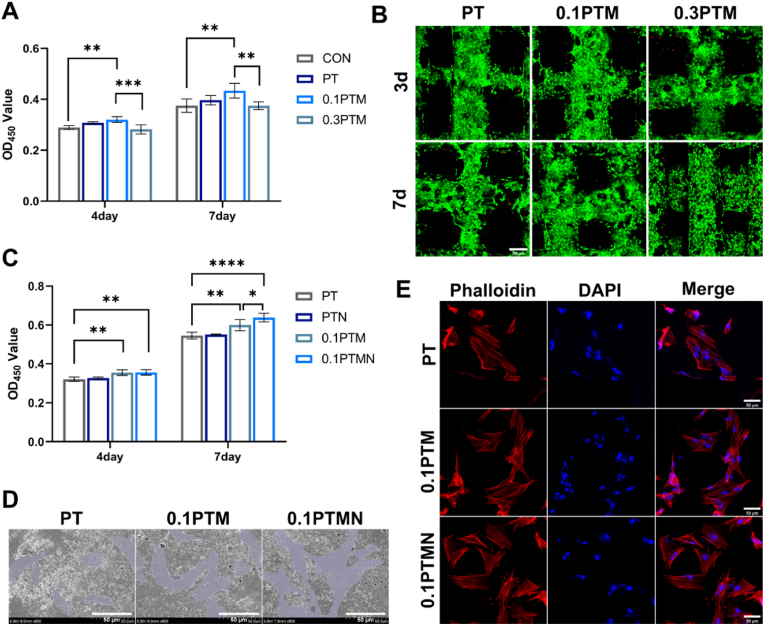
A) CCK-8 assay results of BMSCs cultured on PT, 0.1PTM, and 0.3PTM for 4 days and 7 days. B) Confocal microscopy images and calcein/PI staining of BMSCs cultured on PT, 0.1PTM, and 0.3PTM for 3 days and 7 days. C) CCK-8 assay results of BMSCs cultured on PT, PTN, PTM, and PTMN for 4 days and 7 days. D) SEM images of BMSCs adhering to PT, PTM, and PTMN. E) Confocal microscopy images of BMSCs cultured on PT, PTM, and PTMN stained with rhodamine-phalloidin/DAPI, with red (TRITC-phalloidin) indicating F-actin and blue (DAPI) indicating the nucleus. (*∗p* < 0.05, *∗∗p* < 0.01, *∗∗∗p* < 0.001, *∗∗∗∗p* < 0.0001; "ns" indicates no significant difference, n = 3, error bars represent the mean ± standard deviation). (For interpretation of the references to color in this figure legend, the reader is referred to the Web version of this article.)

Biocompatibility plays a pivotal role in determining the suitability of composite scaffolds for both in vitro and in vivo applications. We seeded bone marrow mesenchymal stem cells onto the composite scaffolds and employed the CCK-8 kit to assess their biocompatibility on the 4th and 7th day post-seeding (Fig. 3A). On the fourth day, there was no significant disparity observed in proliferation between the PT scaffold and control group. However, notably higher cell proliferation was recorded for the 0.1PTM scaffold compared to other groups, indicating its substantial promotion of BMSCs' proliferation. This effect was particularly pronounced on the seventh day, suggesting that PLGA/β-TCP scaffold exhibits exceptional biocompatibility while incorporating Nb_2_C MXene to some extent enhances cell growth. However, the proliferative activity of BMSCs was lower on the 0.3PTM scaffold compared to 0.1PTM, indicating a negative effect of excessive Nb_2_C MXene on both activity and proliferation of BMSCs. We hypothesize that Nb_2_C MXene influences cellular activity through two primary mechanisms: the generation of excess reactive oxygen species (ROS) and the direct damage to cell membranes upon contact. Previous studies have indicated a significant correlation between Nb_2_C MXene and both the production and elimination of ROS. When in contact with water, Nb_2_C MXene facilitates the dissociation of water molecules into radical hydroxide groups (·OH), superoxide anions (·O_2_^−^), and hydrogen ions (H^+^) [61]. Not only are these free radicals capable of damaging cell membranes, but excess reactive oxygen species (ROS) can also surpass the oxidative stress threshold of cells, ultimately leading to cell death. Concurrently, the generated free radicals accept electrons to form hydrogen peroxide anions, which subsequently react with protons (H^+^) to produce hydrogen peroxide [62]. This compound then penetrates the cell membrane, contributing further to cellular demise [62]. Another mechanism underlying MXene toxicity involves its strong adhesion to the cell membrane. This interaction results in membrane instability and a loss of cellular integrity through ionic interactions, van der Waals forces, or receptor-ligand binding [62]. We suggests that an excess amount of Nb_2_C MXene may disrupt cellular oxidative-reduction metabolism balance, resulting in decreased cell viability [63]. Subsequently, we evaluated biocompatibility by live/dead cell staining; findings revealed superior growth characteristics for BMSCs cultured along with 0.1 PTM scaffolds (Fig. 3B and Figure S8, S9). The scaffolds were observed to be covered with green fluorescent on the 3rd and 7th day, while only a minimal amount of red fluorescent was detected, further validating the exceptional biocompatibility of the 0.1PTM stent. Considering the mechanical and photothermal properties as well as the impact on cell proliferation, we determined 0.1PTM was selected for subsequent experiments.

Subsequently, we conducted further investigations to assess the impact of near-infrared (NIR) irradiation on the viability and proliferation capacity of bone marrow-derived mesenchymal stem cells (BMSCs). The BMSCs were seeded onto scaffolds and subjected to daily NIR photothermal treatment (808 nm, 1.5 W/cm^2^) for 120 s at a fixed time. Cell viability was evaluated using the CCK-8 assay on the 4th and 7th day. The results revealed that NIR had no significant effect on BMSC viability on day 4. However, on day 7, the cell proliferation of the 0.1PTMN was significantly higher than that of both PT and 0.1PTM, indicating that NIR irradiation combined with gentle thermal treatment not only preserved cell viability but also enhanced cell proliferation (Fig. 3C). After 7 days culture, the morphological changes of the cells on PT, 0.1PTM, and 0.1PTMN were examined using scanning electron microscopy (SEM). BMSCs adhered to the surfaces of all three scaffolds (Fig. 3D). Compared to the PT scaffold, BMSCs exhibited a more densely distributed and elongated cell morphology on the 0.1PTM and 0.1PTMN scaffolds. Immunofluorescence staining for the cell cytoskeleton revealed enhanced pseudopodia formation of BMSCs on 0.1PTM and 0.1PTMN, indicating improved cellular stretching (Fig. 3E).

### 3.4 Influences of the PTM composite scaffolds combined with mild hyperthermia on the osteogenic differentiation of BMSCs

**Fig. 4:**
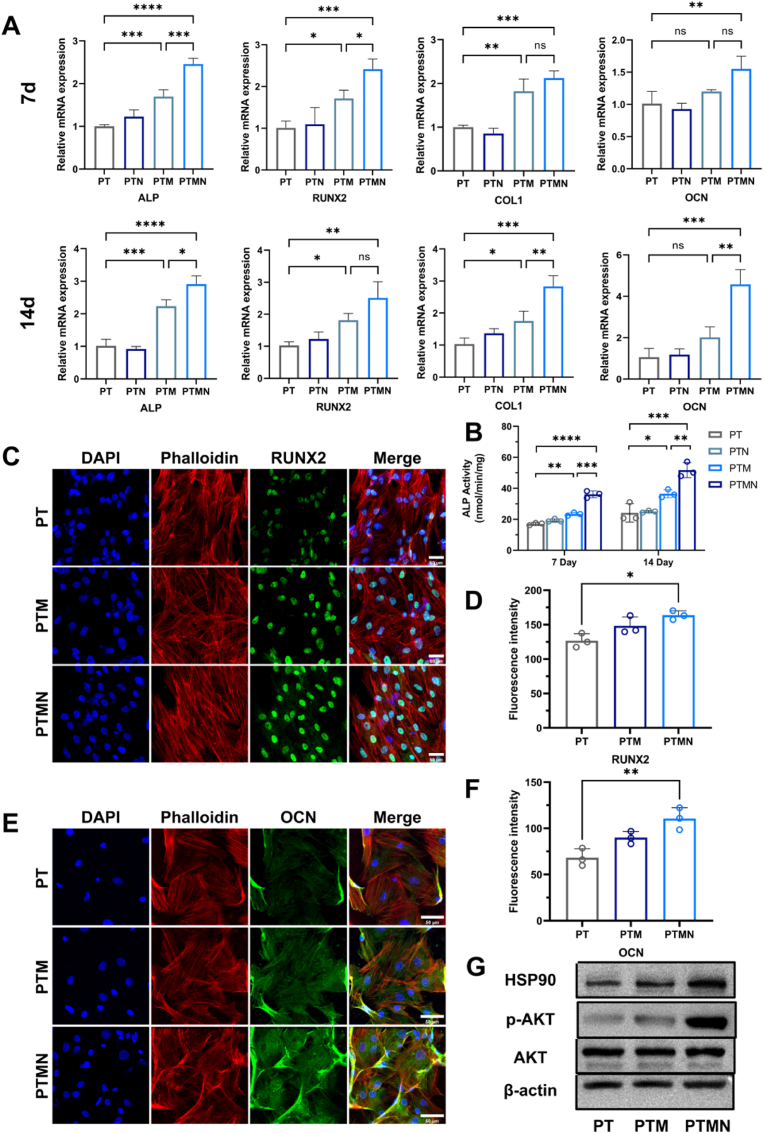
A) Real-time PCR analysis of ALP, RUNX2, COL1, and OCN mRNA expression in BMSCs cultured on different scaffolds at 7 days and 14 days. B) Quantitative analysis of ALP activity in BMSCs cultured on different scaffolds at 7 days and 14 days. C) Immunofluorescence staining of BMSCs cultured on PT, PTM, and PTMN scaffolds for 14 days, with green indicating RUNX2; red (phalloidin) indicating actin; and blue (DAPI) indicating the nucleus. D) Quantitative analysis of RUNX2 fluorescence intensity. E) Immunofluorescence staining of BMSCs cultured on PT, PTM, and PTMN scaffolds for 14 days, with green indicating OCN; red (phalloidin) indicating actin; and blue (DAPI) indicating the nucleus. F) Quantitative analysis of OCN fluorescence intensity. G) Western blot analysis of HSP90, p-AKT, and AKT protein expression in BMSCs cultured on PT, PTM, and PTMN scaffolds for 14 days. (*∗p* < 0.05, *∗∗p* < 0.01, *∗∗∗p* < 0.001, *∗∗∗∗p* < 0.0001; "ns" indicates no significant difference, n = 3, error bars indicate mean ± standard deviation, the qRT-PCR results were expressed as the mean ± error). (For interpretation of the references to color in this figure legend, the reader is referred to the Web version of this article.)

The expression level of osteogenesis-related genes, including Alkaline phosphatase (ALP), type I collagen (COL1), osteocalcin (OCN), and Runt-related transcription factor 2 (RUNX2), were assessed using real-time quantitative polymerase chain reaction (qRT-PCR) after culture on the scaffolds for 7 and 14 days to investigate the regulatory effects of scaffolds combined with photothermal therapy on the osteogenic differentiation of BMSCs (Fig. 4A). At both time points, the PTM and PTMN groups exhibited significantly higher expression levels of these genes compared to the PT and PTN groups. However, no significant difference was observed between the PT and PTN groups, indicating that Nb_2_C MXene combined with photothermal therapy promotes osteogenesis while sole near-infrared light irradiation does not. Furthermore, the expression levels of osteogenic-related genes in the PTM group were higher than those in the PT and PTN groups, suggesting that Nb_2_C MXene alone has inherent osteogenic effects consistent with previous research findings. Among the composite scaffolds, PTMN exhibited the highest expression level of osteogenic-related genes, indicating that the combination of Nb_2_C MXene and mild thermotherapy generated by near-infrared light exposure has a significant osteogenic effect. It is noteworthy that at Day 7, the expression levels of ALP, RUNX2, and COL1 in the PTMN group were significantly higher than those in the other three groups; however, there was no significant difference in OCN expression compared to the PTM group. However, at Day 14, OCN expression in the PTMN group was significantly higher than that in the PTM group. This discrepancy can be attributed to ALP and RUNX2 being early-expressed osteogenic genes while OCN is a late-expressed osteogenic gene. In summary, PTMN effectively stimulates both early and late osteogenic gene expressions. Furthermore, this study evaluated protein expression to assess the osteogenic potential of PTMN scaffold. The quantitative analysis of alkaline phosphatase (ALP) activity (Fig. 4B) demonstrated that ALP activity was highest in the PTMN group consistent with PCR results. The immunofluorescence staining was performed at 14 days post-implantation, and the fluorescence intensities of OCN and RUNX2 were found to be higher on the PTMN scaffold compared to those on the PT and PTM scaffolds (Fig. 4C&E). The quantitative analysis results also confirmed this observation (Fig. 4D&F). In summary, incorporating an appropriate amount of Nb_2_C MXene into the PLGA/β-TCP scaffold can partially enhance osteogenic differentiation, while the mild thermal therapy generated by photo-thermal therapy further augments biomineralization effects. Previous studies have demonstrated that mild thermal stimulation can upregulate heat shock protein expression in cells [41]. Among these proteins, HSP90, a 90 kDa heat shock protein, binds to Akt in vivo forming an Akt-HSP90 complex that stabilizes Akt kinase activity and promotes Akt phosphorylation [44]. To elucidate the osteogenic promoting mechanism of the PTMN scaffold, Western Blot analysis was employed to assess the expression levels of related proteins such as HSP90, Akt, and p-Akt. Notably, compared with other two groups, BMSCs cultured on PTMN scaffold exhibited increased expression levels of p-Akt and HSP90 (Fig. 4G). Based on these findings, we hypothesize that mild thermal stimulation induced by composite scaffolds under near-infrared irradiation can trigger high expression of HSP90; furthermore, HSP90 acts as a stabilizer for p-Akt which activates PI3K/Akt pathway in osteoblasts leading to promotion of osteogenesis and biomineralization. This experimental result suggests that the PTMN composite scaffold possesses a certain degree of potential for clinical application. DBBM, recognized as the most commonly utilized bone graft material in clinical practice, exhibits slow degradation, a low substitution rate, and favorable osteoconductivity. However, it lacks both osteoinductivity and osteogenic activity; consequently, its osteogenic effect is often limited when used in isolation. To enhance the osteogenic effect by providing additional growth factors, some clinicians combine PRP, PRF, or CGF with DBBM. Through relevant in vitro osteogenic experiments, we demonstrated that the PTMN scaffold can upregulate the expression of cytokines associated with osteogenesis. Furthermore, it promotes the osteogenic differentiation of BMSCs and activates the HSP90/PI3K/AKT signaling pathway to facilitate bone defect repair. Thus, this scaffold exhibits certain levels of osteoinductivity.

### 3.5 PTM composite scaffolds + NIR promotes angiogenesis in vitro

**Fig. 5:**
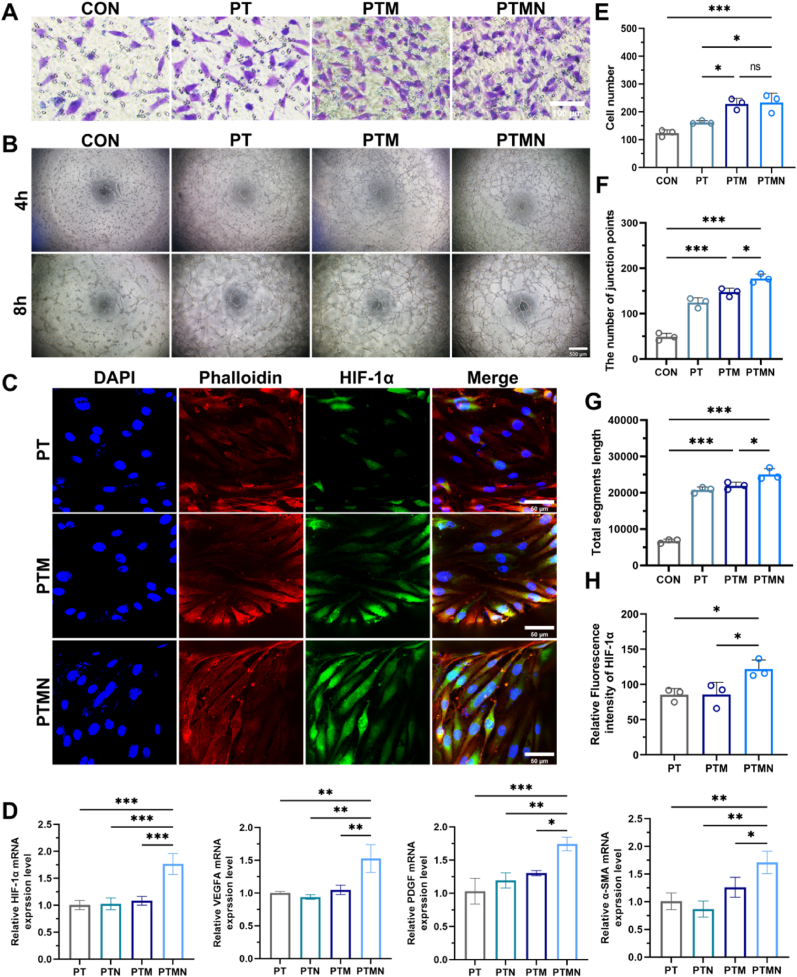
Determination of angiogenic capacity of HUVECs. A) Image of Transwell assay. B) Optical microscope images of tube formation experiments. C) Immunofluorescence staining of HUVECs cultured on different scaffolds for 3 days, with green indicating HIF-1α; red (phalloidin) indicating actin; and blue (DAPI) indicating the nucleus. D) Quantitative real-time PCR analysis of HIF-1α, VEGFA, PDGF, and α-SMA mRNA expression in HUVECs cultured on PT, PTM, and PTMN scaffolds for 3 days. E) Quantitative analysis of the number of cells migrating through the Transwell. F) Quantitative analysis of the number of junction points in the tube formation experiment. G) Quantitative analysis of the total tube length in the tube formation experiment. H) Quantitative analysis of the intensity of HIF-1α immunofluorescence. (*∗p* < 0.05, *∗∗p* < 0.01, *∗∗∗p* < 0.001, *∗∗∗∗p* < 0.0001; "ns" indicates no significant difference, n = 3, error bars indicate mean ± standard deviation, the qRT-PCR results were expressed as the mean ± error). (For interpretation of the references to color in this figure legend, the reader is referred to the Web version of this article.)

Vascularization and the establishment of a new vascular network represent the initial and pivotal steps in bone formation, with blood supply being a major determinant of its prognosis. The vascular network not only facilitates material transport during bone formation but also orchestrates angiogenesis-osteogenesis coupling through intricate signal molecules regulation mechanisms, thereby modulating the process of bone formation. To investigate the impact of the composite scaffold on angiogenesis, we used trans-well assay to assess their influence on endothelial cell migration. As Fig. 5A, Nb_2_C MXene can induce endothelial cell migration. No significant difference was observed between the PTM group and PTMN group, suggesting that mild thermal stimulation triggered by near-infrared irradiation does not further enhance endothelial cell migration (Fig. 5E). To evaluate the angiogenic potential of each scaffold, we conducted a tube formation assay (Fig. 5B). After 4 h of culture, endothelial cells migrated and gradually organized into bifurcation nodes and network structures across all groups (PT, PTM, and PTMN). Following 8 h of culture, there was a notable increase in tubular network structures. Quantitative analysis revealed that compared to other groups, the PTMN group exhibited more complete and structurally intact tubular networks while significantly promoting tube formation (Fig. 5F and G). The expression of HIF-1α, a cellular factor associated with angiogenesis, was assessed using an immunofluorescence assay to detect protein levels on the endothelial cells seeded on the scaffolds in each experimental group (Fig. 5C). Subsequently, quantitative analysis was conducted (Fig. 5H), revealing a significant increase in fluorescence intensity of HIF-1α in the PTMN group compared to both the PT and PTM groups. Additionally, PCR detection showed that the expression of angiogenesis-related genes (HIF-1α, PDGFA, VEGFA, α-SMA) in HUVECs cultured on PTMN was significantly higher than that of the other three groups (Fig. 5D). Based on the experimental results presented above, we conclude that the different functional components of the PTMN scaffold promote angiogenesis at various stages of vascular formation respectively. The PTMN composite scaffold primarily utilizes Nb_2_C MXene to enhance the proliferation and migration of endothelial cells. This mechanism may be associated with the low-dose nitric oxide (NO) release discussed earlier. The Trans-well assay results indicate that the effect of near-infrared irradiation on endothelial cell migration is limited. However, the process of angiogenesis is not only related to the migratory ability of endothelial cells, but also closely associated with their tube-forming differentiation ability. Tube formation experiment, PCR and immunofluorescence analyses demonstrate that photothermal therapy can directly upregulate the expression of angiogenesis-related cytokines and HSP90, promoting endothelial cell function, thereby promoting angiogenesis.

### 3.6 Research on the mechanism of PTMN composite stent promoting angiogenesis

**Fig. 6:**
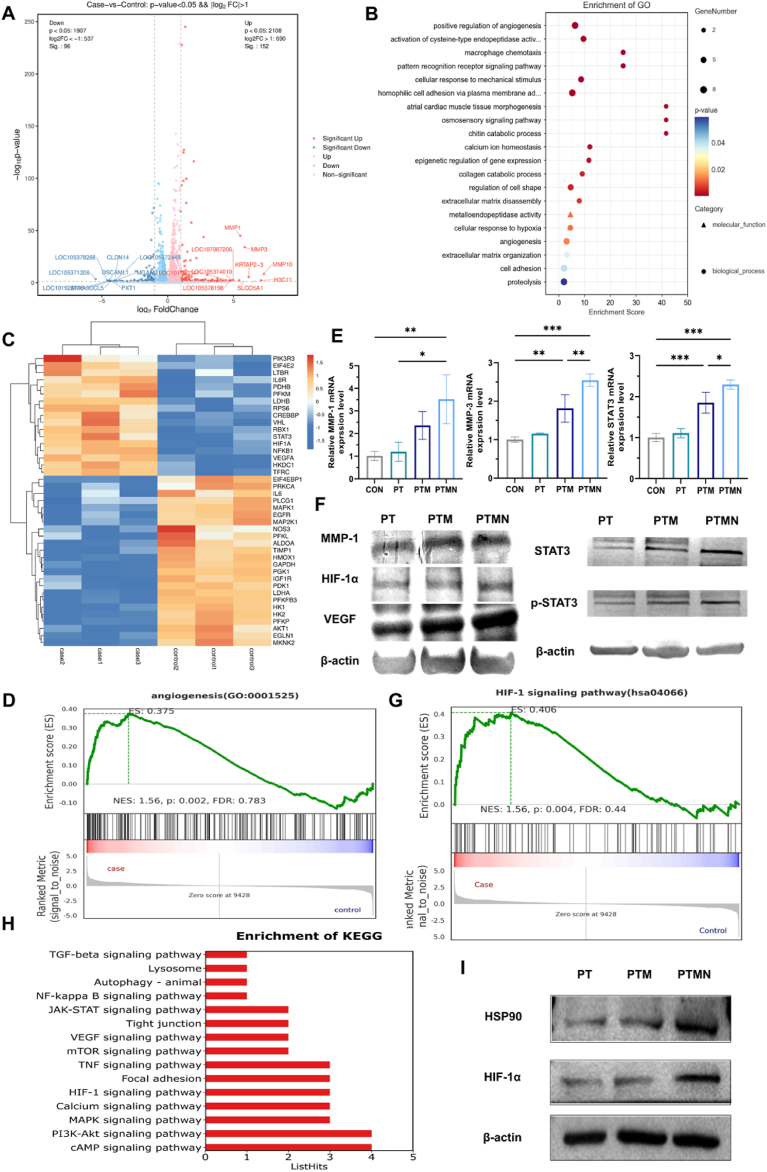
Transcriptome analysis of HUVECs. A) Volcano plot of differentially expressed genes in HUVECs cultured on PTMN scaffolds. B) Bubble plot of GO enrichment analysis of HUVECs [n = 3, adjusted p-value (q-value) <0.5]. C) Heatmap analysis of HUVECs, red: upregulated expression; blue: downregulated expression. (96 downregulated genes, 152 upregulated genes). [n = 3, adjusted p-value (q-value) <0.5]. D) Enrichment analysis of angiogenesis-related processes in GSEA of HUVECs. E) Real-time PCR expression analysis of MMP-1, MMP-3, and STAT3 mRNA in HUVECs cultured on different scaffolds for 3 days. F) Western blot results of MMP-1, HIF-1α, VEGF, STAT3, and p-STAT3 in HUVECs cultured on different scaffolds for 3 days. G) Enrichment analysis results of the HIF-1 pathway in GSEA of HUVECs. H) KEGG pathway enrichment analysis results of HUVECs. I) Western blot results of HSP90 and HIF-1α in HUVECs cultured on different scaffolds for 3 days. (*∗p* < 0.05, *∗∗p* < 0.01, *∗∗∗p* < 0.001, *∗∗∗∗p* < 0.0001; "ns" indicates no significant difference, n = 3, the qRT-PCR results were expressed as the mean ± error). (For interpretation of the references to color in this figure legend, the reader is referred to the Web version of this article.)

Controlled inflammatory response and subsequent angiogenesis are the primary components of early-stage bone defect repair. The degree of inflammation and its controllability, as well as the speed and quality of angiogenesis, are crucial factors determining the efficacy of bone defect repair. Previous experimental findings have demonstrated that the PTMN scaffold can effectively enhance pro-angiogenic properties and promote bone regeneration by upregulating the expression of HIF-1α, VEGF, α-SMA and PDGF. To further investigate the underlying mechanism through which the PTMN scaffold promotes angiogenesis, we performed RNA-seq analysis on HUVECs cultured on both PT and PTMN scaffolds three days after implantation. Correlation analysis revealed a strong correlation between the PT group and the PTMN group (Fig. S10). Based on Bayesian methods (fold change ≥2 and P < 0.05), we identified 248 differentially expressed genes (DEGs) when comparing the PT group with the PTMN group, including 96 down-regulated genes and 152 up-regulated genes, as depicted in volcano plot (Fig. 6A). GO enrichment analysis (Fig. 6B) and heatmap (Fig. 6C) reveal that, compared to the PT group, the PTMN group exhibits significantly upregulated genes associated with angiogenesis, (including MMP-1, MMP-3, MMP-10; STAT3; HIF-1α; and VEGF). Radar chart (Fig. S11) and GSEA enrichment analysis also has similar findings (Fig. 6D). Moreover, these gene expression patterns were validated using qRT-PCR (Fig. 6E) and confirmed at the protein level through Western Blot analysis (Fig. 6F). KEGG pathway enrichment analysis further demonstrates a close association of all these genes with the HIF-1 pathway (Fig. 6G and H). HIF-1 is a heterodimer composed of HIF-1α and HIF-1β subunits [64]. The expression of HIF-1α is induced in response to oxidative stress or hypoxia [65]. Activation of HIF-1 leads to nuclear translocation and binding to hypoxia-responsive elements, thereby transcribing genes associated with angiogenesis [66]. STAT-3 is a transcription factor that can be phosphorylated at tyrosine 705 in response to various cytokines [67] and regulate the expression of downstream cytokines, including VEGF which is related to angiogenesis. We hypothesize that the remarkable angiogenic effect of PTMN stent can be attributed to the upregulation of MMP expression and activation of the HIF-1 pathway: The MMP family plays a pivotal role in degrading the extracellular matrix, which is responsible for remodeling processes. Upregulation of MMP expression expedites basement membrane degradation and subsequent release of endothelial cells, thereby initiating early-stage angiogenesis during bone defect repair. Activation of the HIF-1 pathway enhances HIF-1α and STAT-3 expression, resulting in increased VEGF expression as a downstream angiogenic factor, facilitating blood vessel formation, maturation, and differentiation. Moreover, both HIF-1α and STAT-3 function as receptors for HSP90 [68]. In our previous studies, we have demonstrated that cooperative near-infrared light irradiation of Nb_2_C MXene in BMCS induces mild thermal therapy, which activates the PI3K-AKT pathway and subsequently upregulates HSP90 expression. To confirm the alterations in HSP90 expression and its interaction with HIF-1α in HUVECs, Western blot analysis was performed (Fig. 6I), revealing an increase in both HSP90 and HIF-1α levels. These findings suggest that the PTMN scaffold can activate the HIF-1α pathway while synergistically promoting angiogenesis-osteogenesis coupling through cooperation with the HSP90/PI3K/AKT pathway. Moreover, it is likely that controlled inflammatory response during early-stage bone defects leads to elevated IL-6 expression along with upregulated IL-6R induced by PTMN scaffold action, thereby further reinforcing the positive feedback loop involving IL-6/STAT-3/HIF-1α and facilitating angiogenesis.

**Fig. 7:**
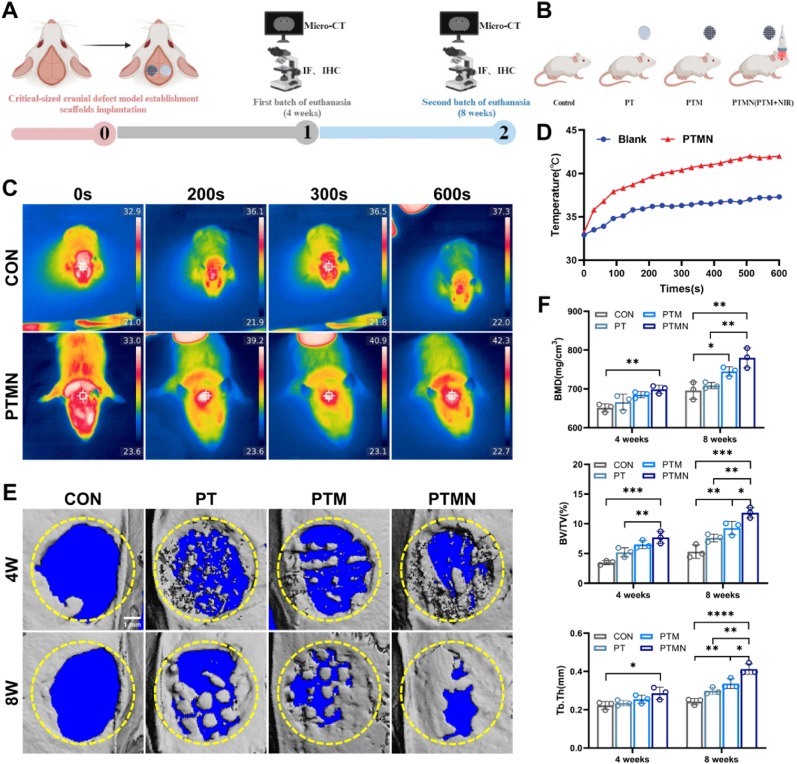
The photothermal effects and bone regenerative ability of different scaffolds in a rat cranial defect model analyzing by Micro-CT. A) Schematic diagram for the analysis of bone regeneration. B) Group specification. C) Real-time near-infrared thermal images of the blank group and PTMN group under in vivo near-infrared radiation (1.5 W cm^−2^) for 10 min. D) Corresponding temperature-time curves. E) Micro-CT images of the bone defect at 4 weeks and 8 weeks post-operation. F) Quantitative analysis of the bone defect by micro-CT, including BMD, BV/TV, and Tb.Th. (*∗p* < 0.05, *∗∗p* < 0.01, *∗∗∗p* < 0.001, *∗∗∗∗p* < 0.0001; "ns" indicates no significant difference, n = 3, error bars represent the mean ± standard deviation; scale bar = 1 mm).

### 3.7 In vivo osteogenesis

In order to investigate the potential of PTMN for effective repair of critical-sized bone defects in vivo, we established a rat cranial bone critical defect model to evaluate its in vivo osteogenic ability (Figs. S12, 7A, 7B). Twenty SD male rats were randomly divided into four groups: blank control group, PT group, PTM group, and PTM + NIR (PTMN) group. The stent was implanted in the rats, and the PTMN group underwent irradiation with 808 nm NIR at a power density of 1.5 w/cm2 for 600 s every three days. The photothermal effect during near-infrared irradiation on the bone defect was monitored using an infrared thermal imager (Fig. 7C). As depicted in Fig. 7D, when the power density reached 1.5 w/cm^2^, there was a gradual increase in temperature from around 33 °C to approximately 40 °C within 250 s with slight fluctuations observed specifically in the PTMN group. In contrast, the blank control + NIR group exhibited a much slower temperature rise and even after 600 s, the highest temperature recorded remained below 38 °C. Therefore, it can be concluded that the set parameters for the PTMN stent can effectively achieve thermal stimulation of deep tissues. Previous studies have demonstrated that the majority of new bone formation occurs during the 4th and 8th weeks after surgery. Therefore, we utilized micro-CT to observe cranial bone defect healing in rats after scaffolds implantation at both 4th and 8th week post-surgery (Fig. 7E). In the control group, extensive bone defects were observed at both time points with minimal new bone formation detected; conversely, the PTMN group exhibited rapid healing of the defect site with evident new bone growth surrounding and within center of the defects. We quantitatively analyzed parameters such as bone volume (BV/TV), density (BMD), and trabecular thickness (Tb.Th) for each group (Fig. 7F). Compared to other groups, significant increases were noted in BV/TV, BMD, and Tb.Th levels within the PTMN group further demonstrating its exceptional potential for regenerating critical bone defects.

**Fig. 8:**
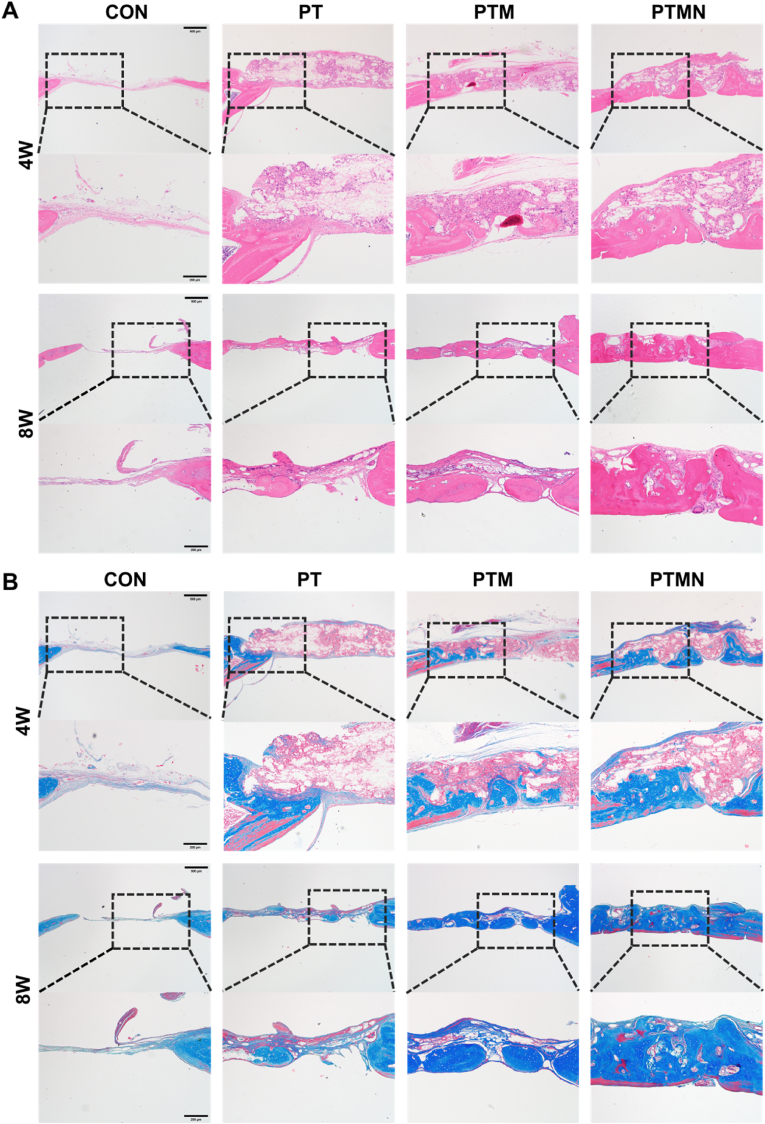
Bone regenerative ability of different scaffolds in a rat cranial defect model analyzed by histology. A) H&E staining images of the bone defect at 4 weeks and 8 weeks post-operation in each group. B) Masson trichrome staining images of the bone defect at 4 weeks and 8 weeks post-operation in each group. (scale bar = 200 μm).

The histological assessment and quantitative analysis of bone regeneration for each group were conducted at 4th and 8th week using hematoxylin and eosin (H&E) staining, as well as Masson trichrome staining. The H&E staining images revealed extensive new bone regeneration in the PTMN group, accompanied by abundant matrix formation within the defect area (Fig. 8A). At 4 weeks, the PTM group exhibited only a limited amount of newly formed bone, whereas in the PTMN group, new bone was observed both at the periphery and center of the defect. By 8 weeks, substantial amounts of fibrous connective tissue were still present in both the control and PT groups; however, partial repair was evident in the PTM group and nearly complete repair was observed in the PTMN group based on H&E results. Consistent with these findings, Masson trichrome staining images revealed scattered collagen matrices in the control and PT groups but dense and mature collagen matrices in the PTMN group (Fig. 8B). Quantitative analysis demonstrated significantly higher collagen content in the PTMN group compared to that in the PTM group, indicating robust bone formation activity (Figs. S15 and S16). The results of in vivo experiments further validated the clinical application potential of PTMN scaffolds, which exhibited excellent photothermal stability and effective bone defect repair in a rat model with critical-sized cranial defects. However, it is important to note that the skin of rats is relatively thin, resulting in a limited requirement for near-infrared light penetration. In previous research conducted by our group, we utilized pig skin samples measuring 2 mm, 5 mm, and 7 mm to assess the penetration of near-infrared light through the skin [69]. This investigation aimed to demonstrate the feasibility of photothermal materials for clinical applications.

**Fig. 9:**
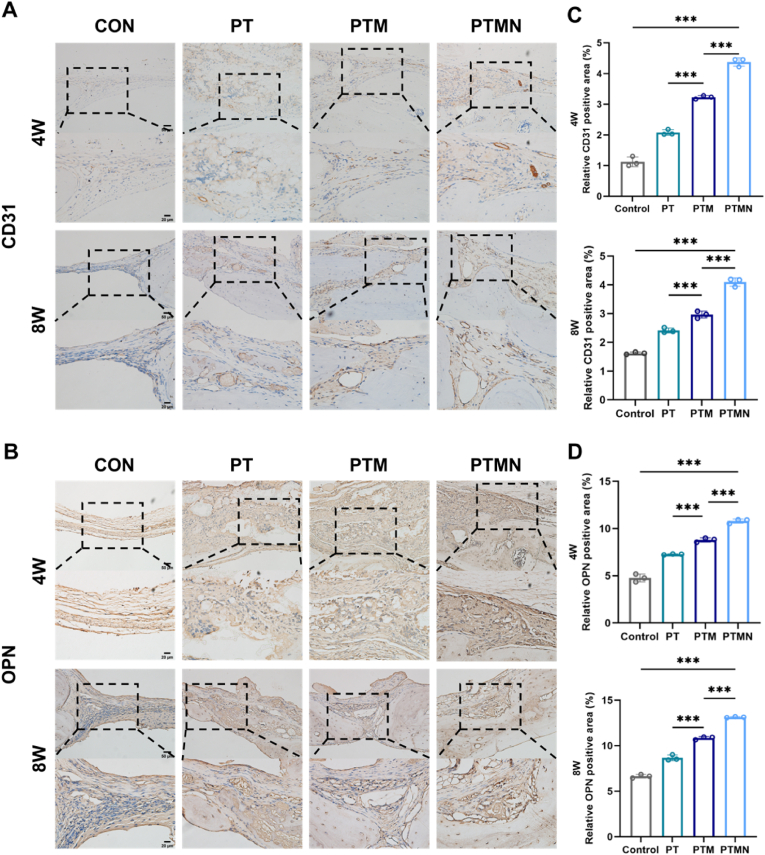
Immunohistochemical evaluation of angiogenesis and bone regeneration effects. A) CD31 immunohistochemical staining of tissue sections from the bone defect site at 4 weeks and 8 weeks after stent implantation. B) OPN immunohistochemical staining of tissue sections from the bone defect site at 4 weeks and 8 weeks after stent implantation. C) Quantitative analysis of CD31 immunohistochemical staining at 4 weeks and 8 weeks. D) Quantitative analysis of OPN immunohistochemical staining at 4 weeks and 8 weeks. (*∗p* < 0.05, *∗∗p* < 0.01, ∗∗∗p < 0.001, *∗∗∗∗p* < 0.0001; "ns" indicates no significant difference, n = 3, error bars represent the mean ± standard deviation; 20 × : scale bar = 50 μm; 40 × : scale bar = 20 μm).

Then, we performed immunohistochemical staining, and the results showed that the number and staining intensity of CD31 (Fig. 9D&F) and OPN (Fig. 9B&D) positive cells in the PTMN group were the largest at 4 weeks and 8 weeks, further confirming the excellent angiogenic bone regeneration performance of the PTMN scaffold.

**Fig. 10:**
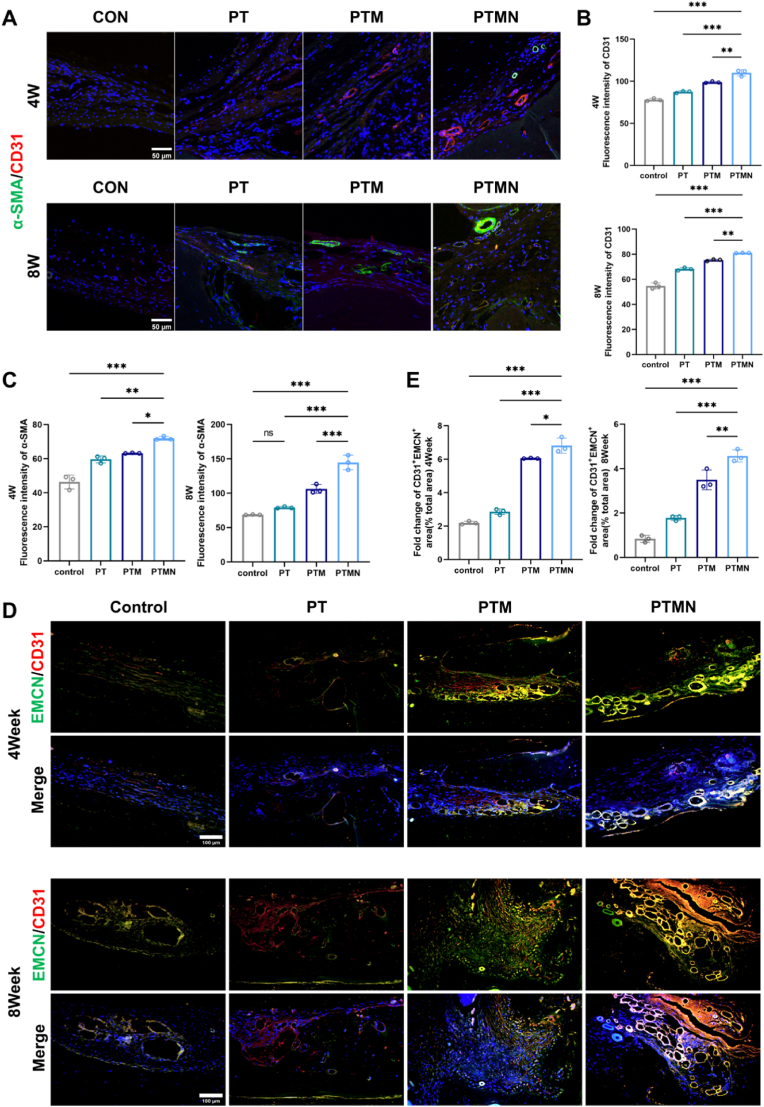
Histological evaluation of angiogenesis and bone regeneration effects. A) α-SMA/CD31 coimmunofluorescence staining of tissue sections from the bone defect site at 4 weeks and 8 weeks after stent implantation. Green represents α-SMA, red represents CD31, and blue (DAPI) represents the nucleus. B) Quantitative analysis of CD31 immunofluorescence staining at 4 weeks and 8 weeks. C) Quantitative analysis of α-SMA immunofluorescence staining at 4 weeks and 8 weeks. D) H-type vascular morphology: α-SMA/CD31 coimmunofluorescence staining of tissue sections from the bone defect site at 4 weeks and 8 weeks after stent implantation. Green represents EMCN, red represents CD31, and blue (DAPI) represents the nucleus. E) Quantitative analysis of the proportion of H-shaped vessels at 4 weeks and 8 weeks. (*∗p* < 0.05, *∗∗p* < 0.01, ∗∗∗p < 0.001, *∗∗∗∗p* < 0.0001; "ns" indicates no significant difference, n = 3, error bars represent the mean ± standard deviation). (For interpretation of the references to color in this figure legend, the reader is referred to the Web version of this article.)

In vitro experiments have shown that the significant osteogenic capacity of the PTMN scaffold is partly based on its strong angiogenic potential. Therefore, we conduct the immunofluorescence staining and quantitative analysis of CD31 and α-SMA in each group to show the angiogenic ability. The results showed that the density of new and mature blood vessels in the defect area of the PTMN group was significantly higher than that of the other groups (Fig. 10A–C). As previously mentioned, extensive literature confirms that in all vascular subtypes, H-type vessels surrounding CD31 and EMCN-highly expressed vessels are densely populated with bone progenitor cells capable of differentiating into osteoblasts and osteocytes. These cells can facilitate the coupling between angiogenesis and osteogenesis through signaling pathways such as Notch signaling and the HIF-1α pathway [[70], [71], [72]]. we used co-immunofluorescence staining and fluorescence intensity quantitative analysis to further evaluate the expression of H-type vessels in each group at 4 weeks and 8 weeks (Fig. 10D and E). The proportion of H-type vessels in the PTMN composite scaffold group was much higher than that of the other three groups. The increased proportion of H-type vessels in the bone defect area, along with the upregulation of the HIF-1 pathway, can enhance angiogenic-osteogenic coupling. This finding suggests that the PTMN scaffold possesses a greater potential for vascularized bone regeneration.

## 4 Conclusion

The photothermal PLGA/β-TCP/Nb_2_C MXene composite scaffold was fabricated using low-temperature 3D printing technology in this study. Upregulating the expression levels of MMPs facilitates the degradation of the basement membrane and promotes the release of endothelial cells, thereby initiating vascular remodeling. Upon exposure to near-infrared irradiation, this process effectively activates the HIF-1 pathway and enhances the secretion of angiogenesis-related cytokines, consequently promoting angiogenesis at various stages. Furthermore, the coupling of angiogenesis and osteogenesis is enhanced by increasing the proportion of H-type vessels in the bone defect area. Additionally, the scaffold generates a moderate thermal stimulus (40–42 °C), which upregulates the expression of Hsp90 in osteoblasts and endothelial cells. HSP90 functions as a stabilizer for p-Akt, thereby activating the PI3K/Akt pathway to enhance vascularization, bone formation, and biomineralization. Additionally, HIF-1α and STAT3, both of which are signaling molecules within the HIF-1 pathway, also act as receptors for HSP90. Consequently, the synergistic effect of the PI3K/AKT/HSP90 pathway is amplified. Our study provides a new reference for the personalized and precise treatment of large-area bone defects, utilizing 3D printing technology combined with a thermal stimulation-based bone regeneration strategy.

## CRediT authorship contribution statement

**Yi Zhang:** Writing – original draft, Methodology, Investigation. **Mucong Li:** Writing – original draft, Methodology, Investigation. **Hao Zhang:** Writing – review & editing, Software, Investigation. **Jiaqian You:** Writing – review & editing, Validation. **Jing Zhou:** Writing – review & editing, Investigation. **Sicong Ren:** Writing – review & editing, Project administration. **Jian Feng:** Writing – review & editing, Supervision. **Yuzhu Han:** Writing – review & editing, Project administration. **Yidi Zhang:** Writing – review & editing, Project administration. **Yanmin Zhou:** Writing – review & editing, Supervision, Funding acquisition.

## Declaration of competing interest

The authors declare that they have no known competing financial interests or personal relationships that could have appeared to influence the work reported in this paper.

## Data Availability

No data was used for the research described in the article.
