# Supplementary material for: 3D-printed intelligent photothermal conversion Nb2C MXene composite scaffolds facilitate the regulation of angiogenesis-osteogenesis coupling for vascularized bone regeneration
Source: Mater Today Bio. 2025 Mar 8;31:101647. doi: 10.1016/j.mtbio.2025.101647 (PMC11950769; doi:10.1016/j.mtbio.2025.101647)
Supplement: Multimedia component 1 [file mmc1.docx]

**3D-printed intelligent photothermal conversion Nb_2_C MXene composite scaffolds facilitate the regulation of angiogenesis-osteogenesis coupling for vascularized bone regeneration**

**Yi Zhang and Mucong Li, Hao Zhang, Jiaqian You, Jing Zhou, Sicong Ren, Jian Feng, Yuzhu Han, Yidi Zhang^*^ and Yanmin Zhou^*^**

**Yi Zhang^1,2,3^, *Mucong Li* ^1,2^, Author 1; *Hao Zhang*^1,2,4^, Author 2; *Jiaqian You* ^1,2,5^, Author 3; Jing Zhou ^1,2^, Author 4; *Sicong Ren* ^1,2^, Author 5; *Jian Feng* ^1,2^, Author 6; *Yuzhu Han* ^1,2^, Author 7; Yidi Zhang^1,2^* and Yanmin Zhou^1,2^***

**1. Hospital of Stomatology, Jilin University, Changchun 130021, Jilin, China;**

**2. Jilin Provincial Key Laboratory of Tooth Development and Bone Remodeling, Hospital of Stomatology, Jilin University, Changchun 130021, Jilin, China;**

**3. Affiliated Maternal and Child Health Care Hospital of Nantong University, Nantong 226000, Jiangsu, China;**

**4. Department of Stomatology, People's Hospital of Xizang Autonomous Region, Xizang 850000, China;**

**5. Hospital of Stomatology, Guanghua School of Stomatology, Sun Yat-sen University and Guangdong Provincial Key Laboratory of Stomatology, Guangzhou, 510055, Guangdong, China;**

**Yi Zhang and Mucong Li have contributed equally to this work.**

*** Correspondence:**

**Yanmin Zhou**

[**zhouym@jlu.edu.cn**](mailto:zhouym@jlu.edu.cn)

**Appendix S1:Preparation of PLGA/β-TCP scaffolds with different proportions**

The quantities of PLGA and β-TCP necessary for the preparation of PLGA/β-TCP scaffold in various proportions are presented in Table S1. PLGA was dissolved in 4 mL of dichloromethane, followed by the addition of β-TCP particles, which were sonicated for 30 minutes in an ice water bath. Manual stirring for 20 minutes was then performed, and the mixture was loaded into a cylindrical container of 10mm*10mm*40mm. Then, the scaffold was frozen dried for 48 hours to remove water and dichloromethane.

**Table S1.** **The contents of various components of different proportions of PLGA/β-TCP scaffolds**

| Proportions | PLGA (g) | β-TCP (g) |
| --- | --- | --- |
| PLGA/β-TCP (2:1) | 1.33 | 0.67 |
| PLGA/β-TCP (1:1) | 1 | 1 |
| PLGA/β-TCP (1:2) | 0.67 | 1.33 |

**Appendix S2: Surface Topography Observation**

After surface gold plating of the scaffold material, the surface morphology of the scaffold was observed using a scanning electron microscope (SEM, FlexSEM 1000, Hitachi, Japan).

The results are shown in Figure S1. As the inclusion ratio of β-TCP increased, the number of pores in the composite scaffold decreased; however, the surface texture of the scaffold became rougher. At a PLGA/β-TCP ratio of 2:1, the scaffold exhibited a smooth surface with a higher density of pores (1-10um). In contrast, at a PLGA/β-TCP ratio of 1:1, while the surface became rougher, it still retained some um-grade pores that are beneficial for osteoblast proliferation[1-3]. When the ratio was adjusted to PLGA/β-TCP=1:2, β-TCP particles were distinctly visible on the scaffold's surface and um-level pores were notably scarce. Therefore, we conclude that scaffolds with a PLGA/β-TCP ratio of 1:1 possess an optimal combination of surface roughness and pore structure conducive to osteoblast growth.
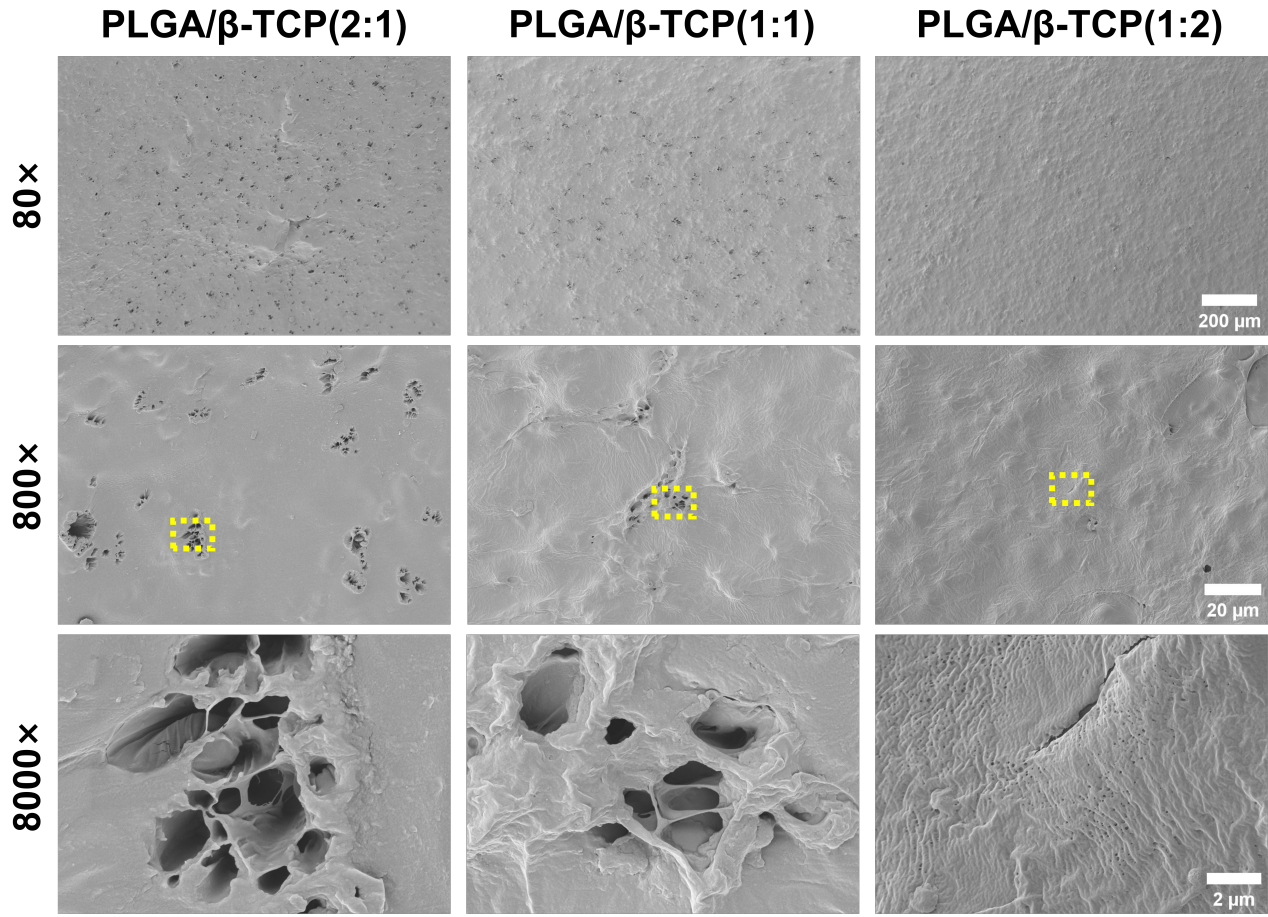


**Figure S1.** Representative SEM of scaffolds surface. The upper row was at 80×, magnified to 800× (middle row); The yellow frame within the middle row was a region of interest at 800×, magnified to 800× (lower row).

**Appendix S3: Porosity Determination**

The porosity of the scaffold was detected using the liquid displacement method. The scaffold was immersed in a certain volume (V_1_) of anhydrous ethanol, and the volume of the scaffold completely immersed was recorded as V_2_. After removing the scaffold, the volume of ethanol at this time was recorded as V_3_. The porosity of the scaffold was calculated using Equation:

Porosity (%) = (V_1_-V_3_)/(V_2_-V_3_) × 100%

The results are shown in Figure S2. The porosity of the composite scaffolds ranges from 60% to 80%, which are favorable for osteogenesis [4, 5]. Additionally, it is observed that the porosity of these composite scaffolds decreases slightly as the inclusion of β-TCP particles increases.


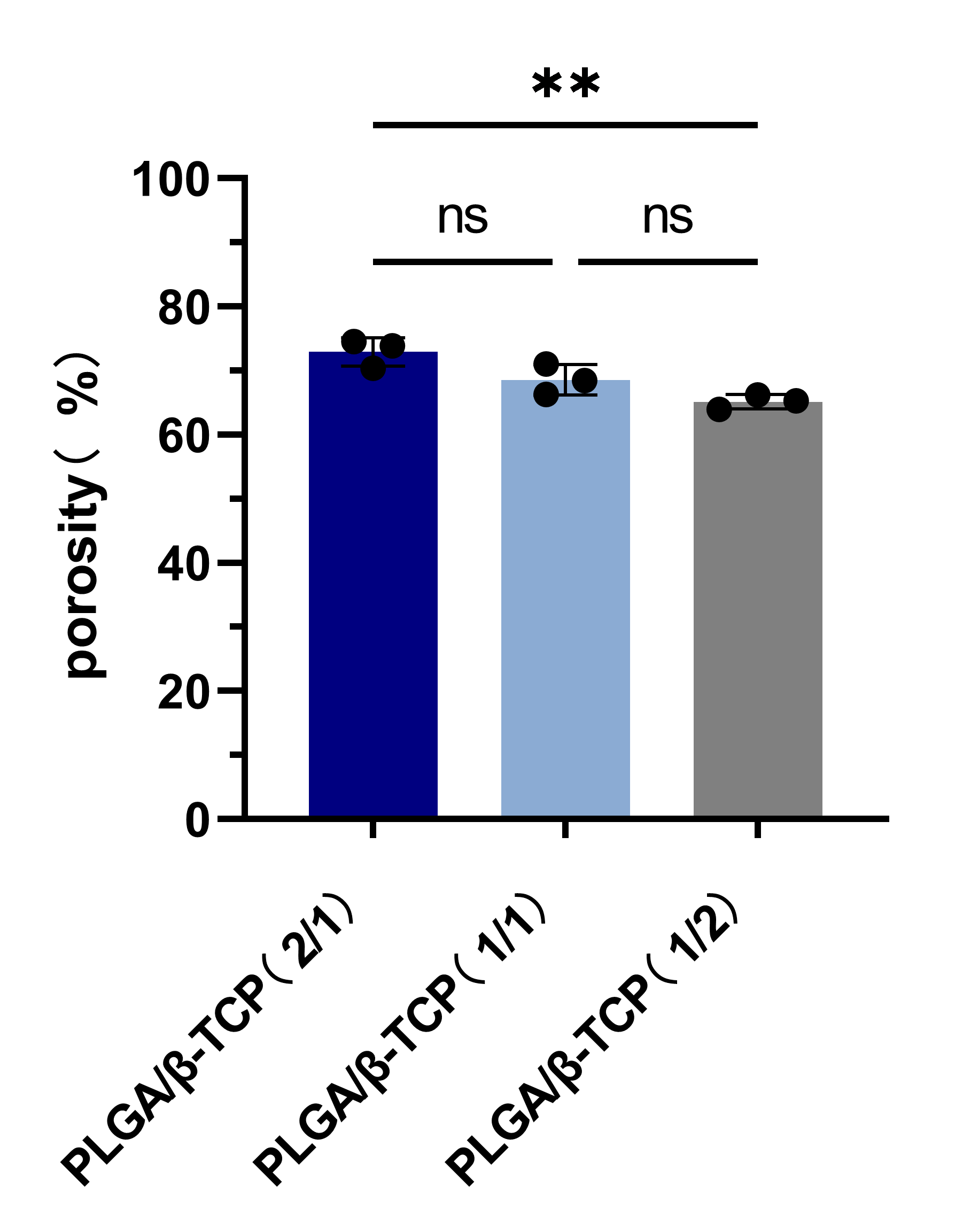


**Figure S2.** Quantitative analysis for the porosity of PLGA/β-TCP (2:1), PLGA/β-TCP (1:1), PLGA/β-TCP (1:2).

**Appendix S4: Mechanical Performance**

The compressive strength of the scaffold was tested using a universal testing machine (SHIMADZU AG-XPLUS10KN, JAPAN) with a capacity of 100 N. The scaffold was prepared into a 10×10×20mm cylindrical shape. The compression speed was 1 mm/min, and the compression distance was stopped when 4 mm remained. The stress-strain curve was drawn to observe the compressive strength of the scaffold, and the modulus of elasticity was the initial slope of the stress-strain curve.

The results are shown in Figure S3. The compressive strength of the three scaffolds is comparable to that of normal bone tissue[6]. Furthermore, it was observed that the compressive strength of the scaffolds increases progressively with a higher incorporation ratio of β-TCP. This finding suggests that adjusting the ratio of β-TCP can enhance the mechanical properties of the composite scaffolds. Notably, a greater proportion of β-TCP correlates with an increase in the compressive strength of the scaffolds.


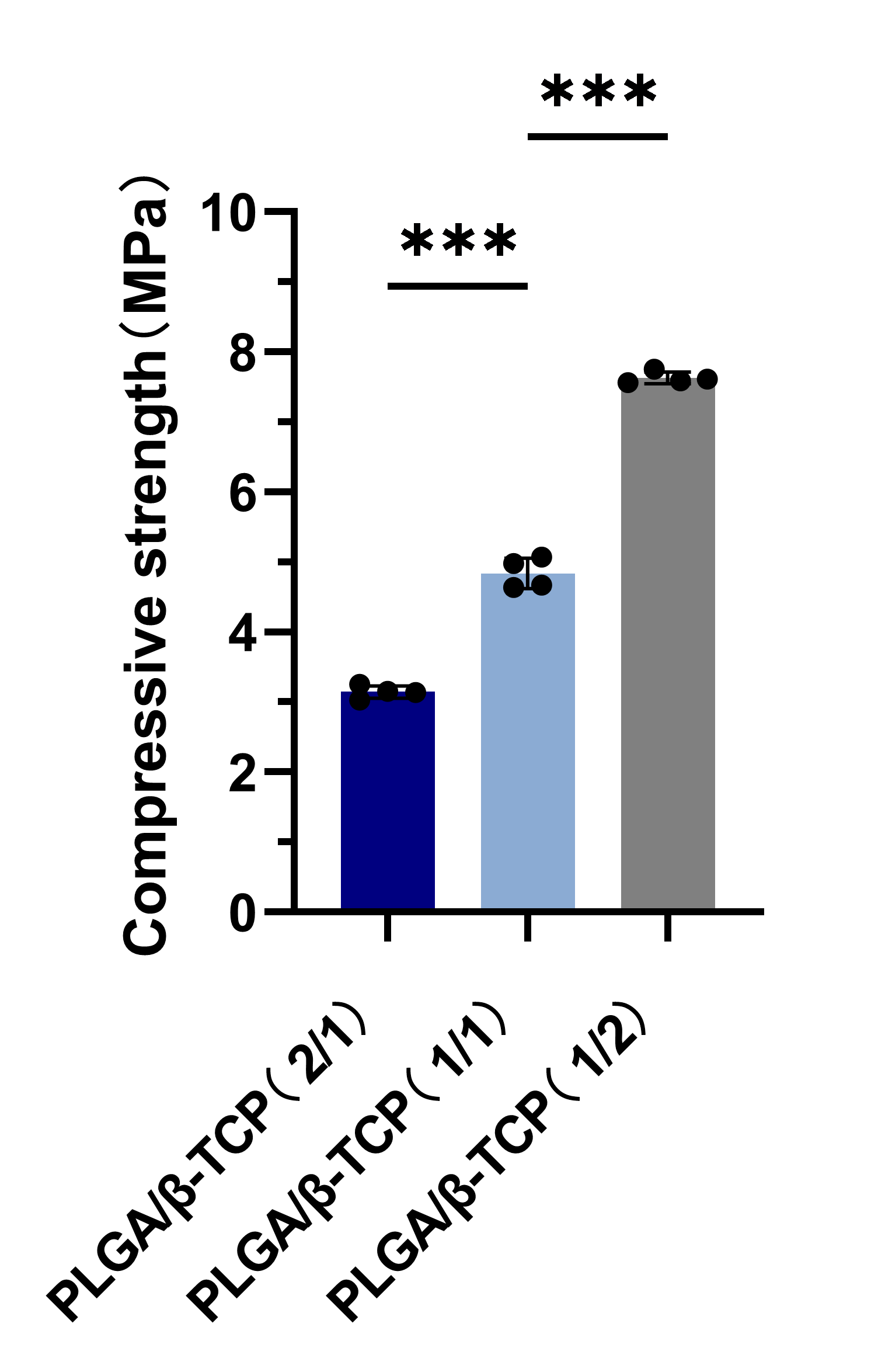


**Figure S3.** quantitative analysis on compressive strength.

**Appendix S5: PH determination and Degradation rate**

The pH value of the degradation medium of each test tube was measured with a pH meter (S20-K, USA) before and at 1-week intervals during degradation with the initial pH value of 7.4.

The results are shown in Figure S4. As the incorporation ratio of β-TCP increases, the reduction in buffer pH during degradation is mitigated, resulting in a more stable environmental pH. This suggests that β-TCP serves a neutralizing function within the composite scaffold. Such functionality not only helps to prevent the self-accelerating degradation effect caused by the accelerated decomposition of ester bonds due to the accumulation of acidic degradation products but also aids in avoiding inflammatory reactions at the implant site that may arise from an acidic environment.


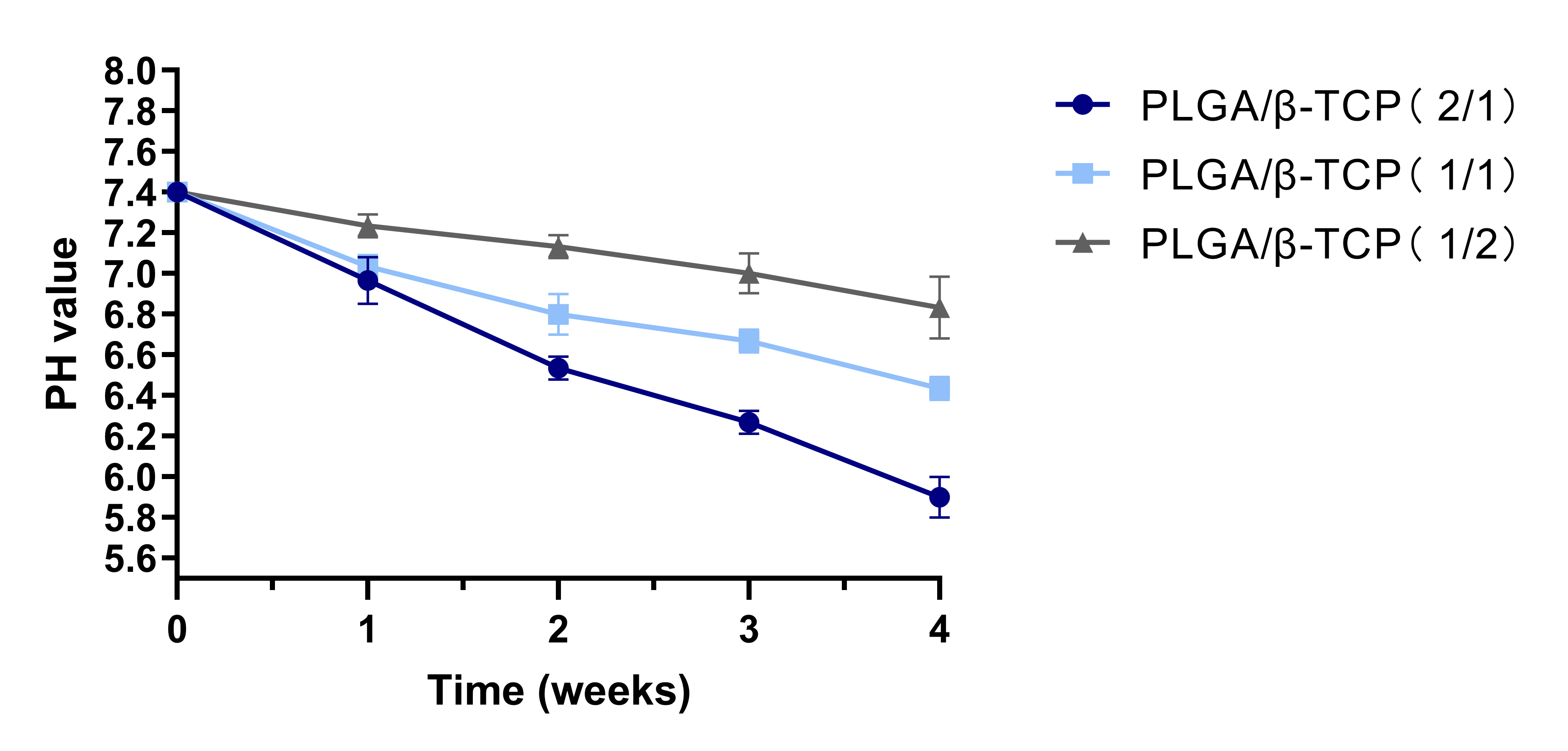


**Figure S4.** pH value-time curve respectively for PLGA/β-TCP (2:1), PLGA/β-TCP (1:1), PLGA/β-TCP (1:2).

The initial weight of the scaffold (W_0_) was measured, and the scaffold (10mm*10mm*4mm) was immersed in 10 mL of sterile PBS solution containing type II collagenase solution (1 U/mL) at 37°C in an incubator for 4 weeks. The weight of the scaffold was measured and recorded (Wt) after freeze-drying and weighing each week. The degradation performance of the stent is evaluated using Equation:

Residual mass ratio (%) = W_t_/W_0_ x 100%

The results are shown in Figure S5. The degradation of the three groups of scaffolds occurred at a relatively slow rate. By the fourth week, the remaining mass of PLGA/β-TCP (2:1) was 95.2 ± 0.36%, that of PLGA/β-TCP (1:1) was 94 ± 0.7%, and that of PLGA/β-TCP (1:2) was 90.8 ± 0.46%. As the incorporation ratio of β-TCP increased, the degradation of the composite scaffolds accelerated; however, their degradation behavior became more stable, with a consistent degradation rate observed during weeks 0 to 4. Due to the accelerated dissolution rate of β-TCP, a higher incorporation ratio leads to a more rapid degradation of the composite scaffold. However, β-TCP can also serve as a neutralizing agent that mitigates the self-accelerating degradation effect caused by an increase in acidic byproducts and a decrease in environmental pH during the degradation process. Therefore, incorporating β-TCP can contribute to achieving a more stable degradation rate for the composite scaffold.
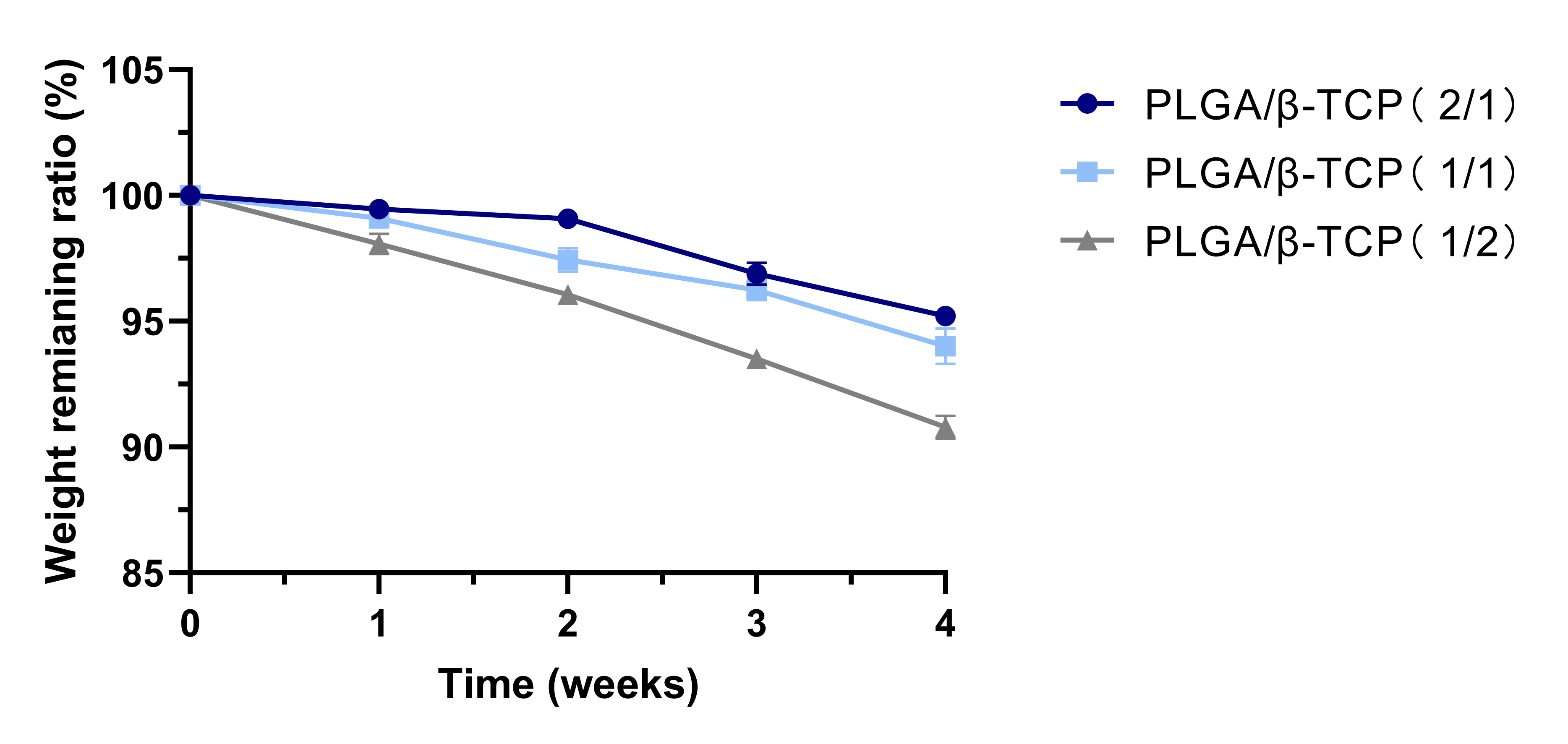


**Figure S5.** degradation rate-time curve respectively for PLGA/β-TCP (2:1), PLGA/β-TCP (1:1), PLGA/β-TCP (1:2).

**Appendix S6: Cell Proliferation and Cell Viability Detection**

The proliferation of BMSCs on the scaffolds was detected using a cell counting kit-8 (CCK-8, NCM Biotech Co. Ltd, China) (n = 3). The PLGA/β-TCP (2:1), PLGA/β-TCP (1:1), and PLGA/β-TCP (1:2) groups of scaffolds (diameter 10 mm, thickness 1.5 mm) were placed in 48-well plates, with 3 replicates in each group. BMSCs were digested and centrifuged, and then seeded onto the scaffolds at a density of 1×10^4^ cells/well with 300 μL of α-MEM complete culture medium per well. After incubation for 4 and 7 days, the old culture medium was replaced with fresh medium, and 10% CCK-8 reaction solution was added to each well at a ratio of 1:10 (culture medium: CCK-8 solution). The mixture was incubated at 37°C in the dark for 1 h, after which 100 μL of the mixture was transferred to a 96-well plate and the OD was measured using an enzyme-linked immunosorbent assay (ELISA) reader (wavelength 450 nm). The results are shown in Figure S6. A comprehensive analysis of the CCK-8 experimental results indicated that the biocompatibility of PLGA/β-TCP (2:1), PLGA/β-TCP (1:1), and PLGA/β-TCP (1:2) was satisfactory; however, the PLGA/β-TCP (1:1) group demonstrated the most significant enhancement effect. Consequently, we selected PLGA/β-TCP (1:1) as the optimal ratio for bio-ink applications.


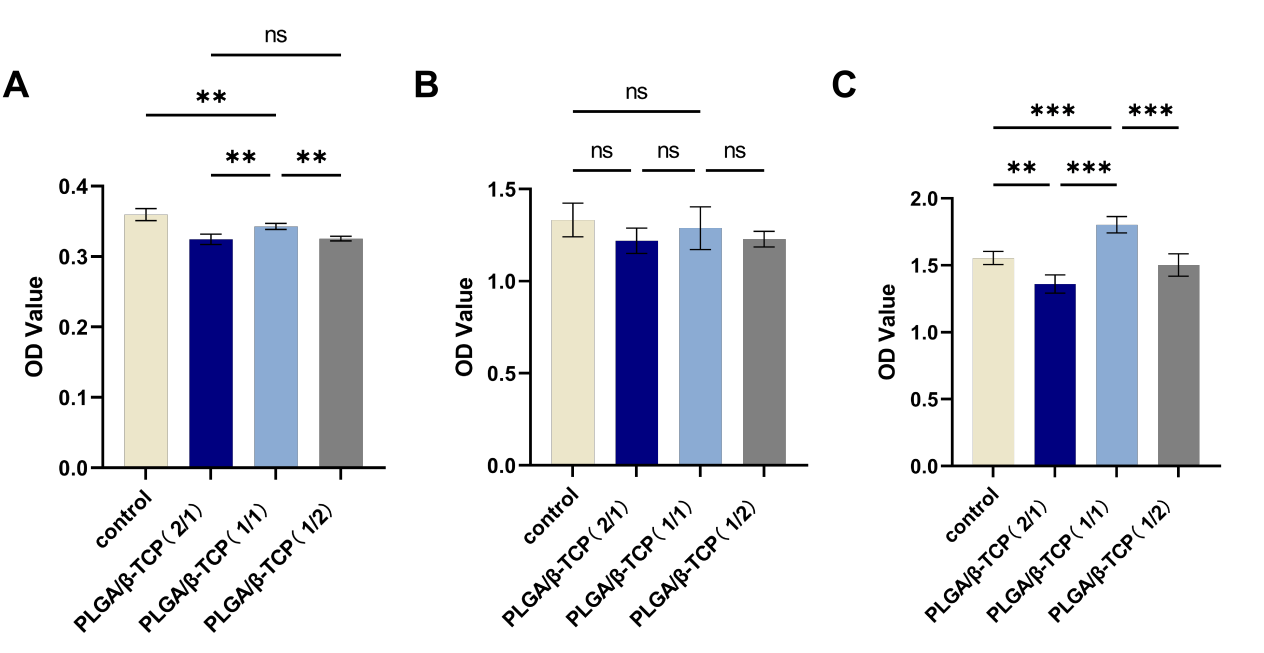


**Figure S6.** CCK-8 assay results of BMSCs cultured on PLGA/β-TCP (2:1), PLGA/β-TCP (1:1), and PLGA/β-TCP (1:2) scaffold for A) 1 day B) 4 days and C) 7 days.

**Table S2.** The usage of RT-qRCT primers for osteogenesis-related genens.

| Genes | Sequences |  |
| --- | --- | --- |
| β-actin | F: 5’ - GGAGATTACTGCCCTGGCTCCTA - 3’ | R: 5’ - GACTCATCGTACTCCTGCTTGCTG - 3’ |
| COL-1 | F: 5’ - GACATGTTCAGCTTTGTGGACCTC - 3’ | R: 5’ - AGGGACCCTTAGGCCATTGTGTA - 3’ |
| OCN | F: 5’ - AAGCAGGAGGGCAATAAGGT - 3’ | R: 5’ - CCGTAGATGCGTTTGTAGGC - 3’ |
| RUNX2 | F: 5’ - CATGGCCGGGAATGATGAG - 3’ | R: 5’ - TGTGAAGACCGTTATGGTCAAAGTG - 3’ |
| ALP | F: 5’- CATCGCCTATCAGCTAATGCACA -3’ | R: 5’- ATGAGGTCCAGGCCATCCAG -3’ |

**Table S3.** The usage of RT-qRCT primers for angiogenesis-related genens.

| Genes | Sequences |  |
| --- | --- | --- |
| GAPDH | F: 5’ - AGAAGGCTGGGGCTCATTTG - 3’ | R: 5’ - AGGGGCCATCAGTCTTC - 3’ |
| VEGFA | F: 5’ - CAGAAGGAGGAGGGCAGAA - 3’ | R: 5’ - GTCTCGATTGGATGGCAGTAG - 3’ |
| PDGFA | F: 5’ - GCAGGAAGCAGGAATGTC - 3’ | R: 5’ - ATACTACAGCGAGGAGGTGTG - 3’ |
| HIF-1α | F: 5’ - AGAAACCACCTATGACCTGCT - 3’ | R: 5’ - CGACTGAGGAAAGTCTTGCTA - 3’ |
| α-SMA | F: 5’ - GACGACGAATCTTCTCAATGG - 3’ | R: 5’ - TGTGTCAGTTTACGATGGCAG - 3’ |

**Table S4.** The usage of RT-qRCT primers for Gene sequencing results verification.

| Genes | Sequences |  |
| --- | --- | --- |
| GAPDH | F: 5’ - AGAAGGCTGGGGCTCATTTG - 3’ | R: 5’ - AGGGGCCATCAGTCTTC - 3’ |
| MMP-1 | F: 5’ -TGACTTTTAAAACATAGTCTATGTTCA - 3’ | R: 5’ - TCTTGGATTGATTTGAGATAAGTCATAGC - 3’ |
| MMP-3 | F: 5’ -GGTTCTCCATTCCTTTGATGGGGGGAAAGA - 3’ | R: 5’ -CTTCCTGGAATTCACATCACTGCCACCACT - 3’ |
| STAT3 | F: 5’ - AAGGAGGCGTCACTTTCACT - 3’ | R: 5’ - AACTTGGTCTTCAGGTATGGG - 3’ |


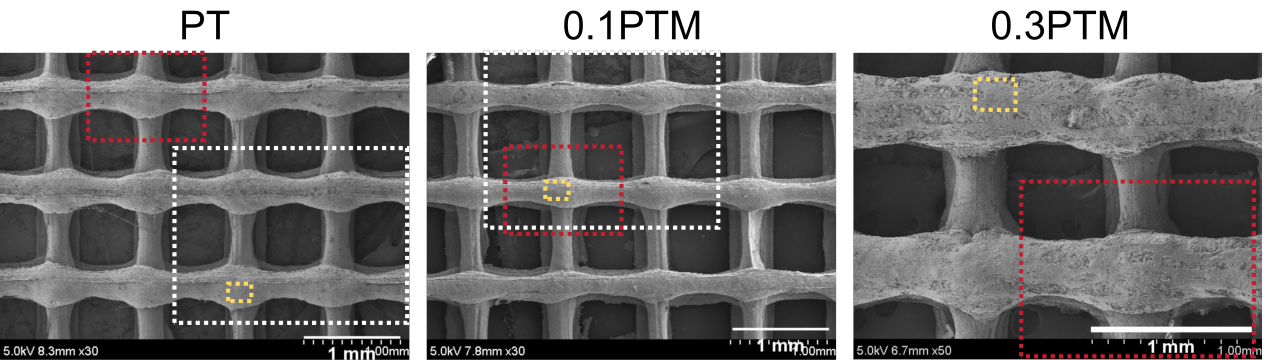


**Figure S7.** SEM thumbnail, a1, b1, c1: white, a2, b2, c2: red, a3, b3, c3: yellow


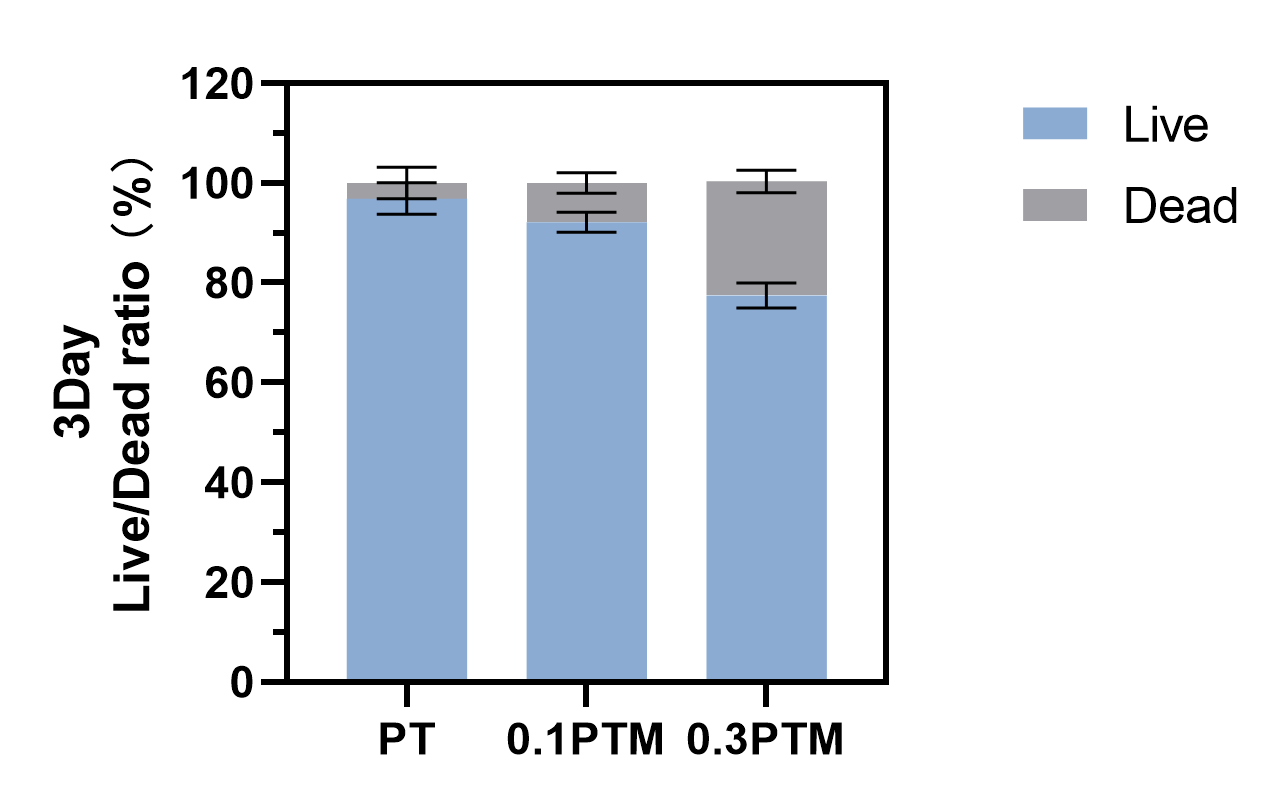


**Figure S8.** Quantitative analysis of live/dead staining of BMSCs. (3 DAY；error bars indicate means ± standard deviations)


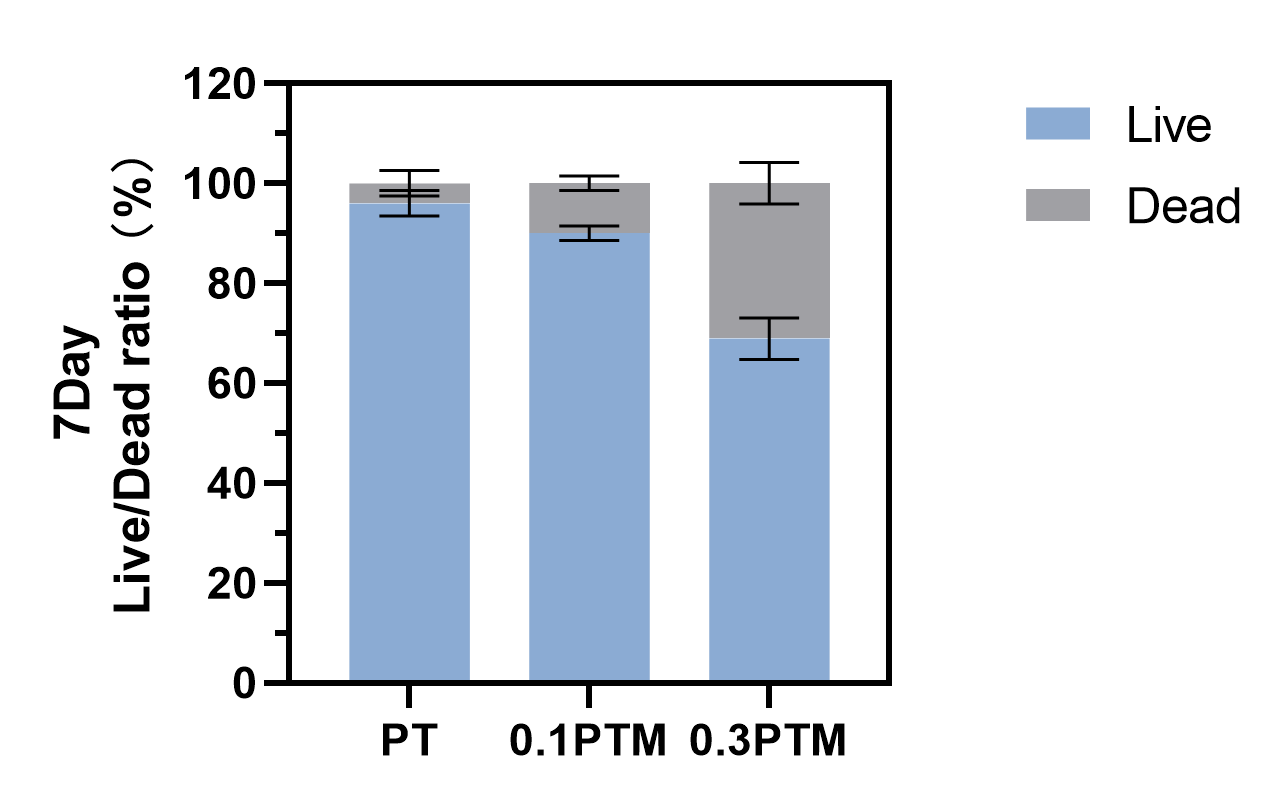


**Figure S9.** Quantitative analysis of live/dead staining of BMSCs. (7 DAY；error bars indicate means ± standard deviations)


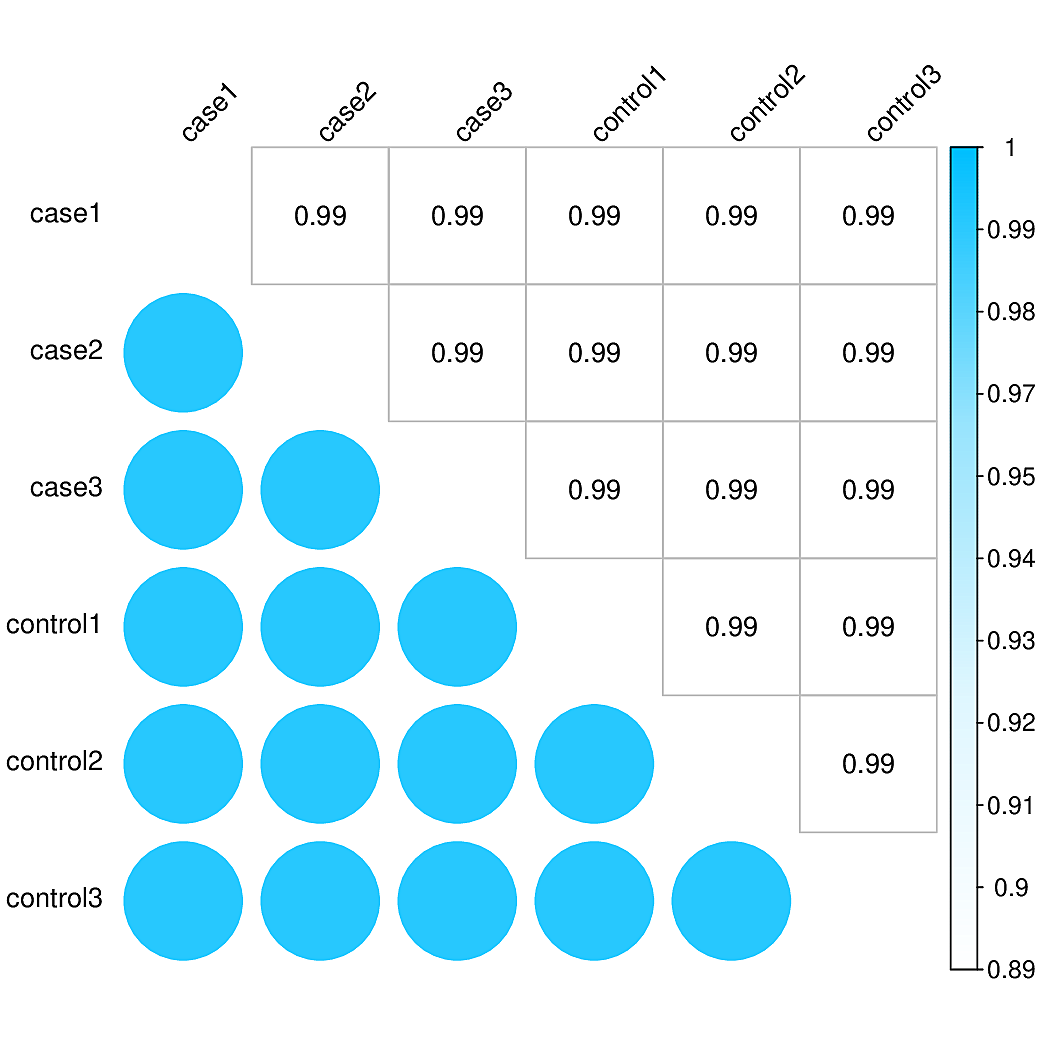


**Figure S10.** Comprehensive Genetic Profiling Analysis of HUVECs


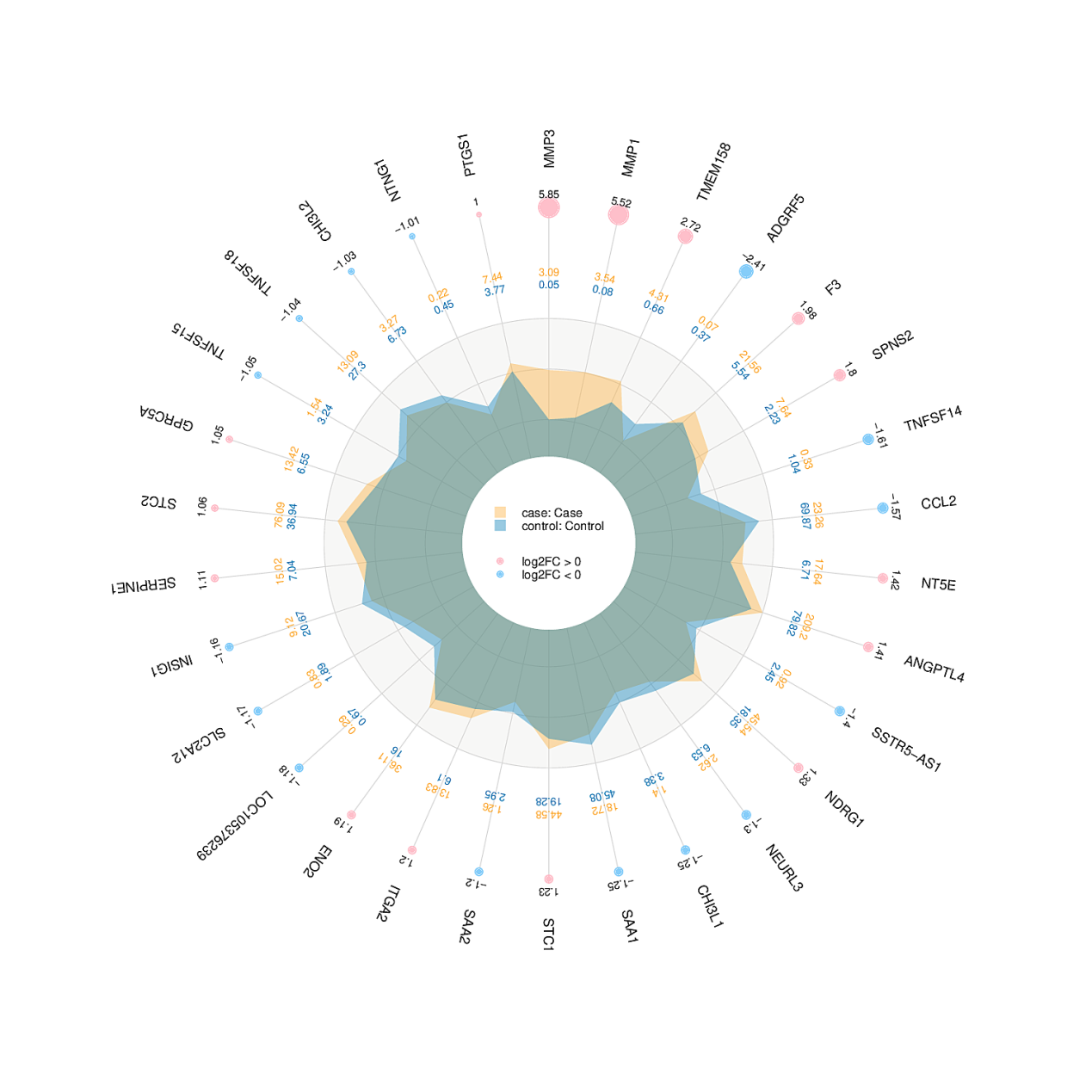


**Figure S11.** Radar chart of HUVECs.


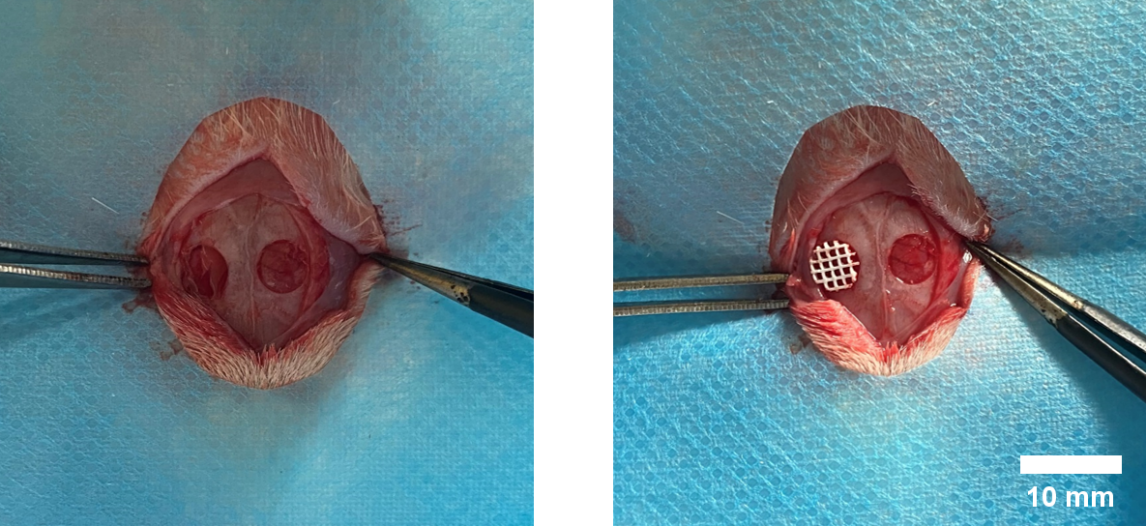


**Figure S12.** Surgical photo of rat skull defect model modeling.


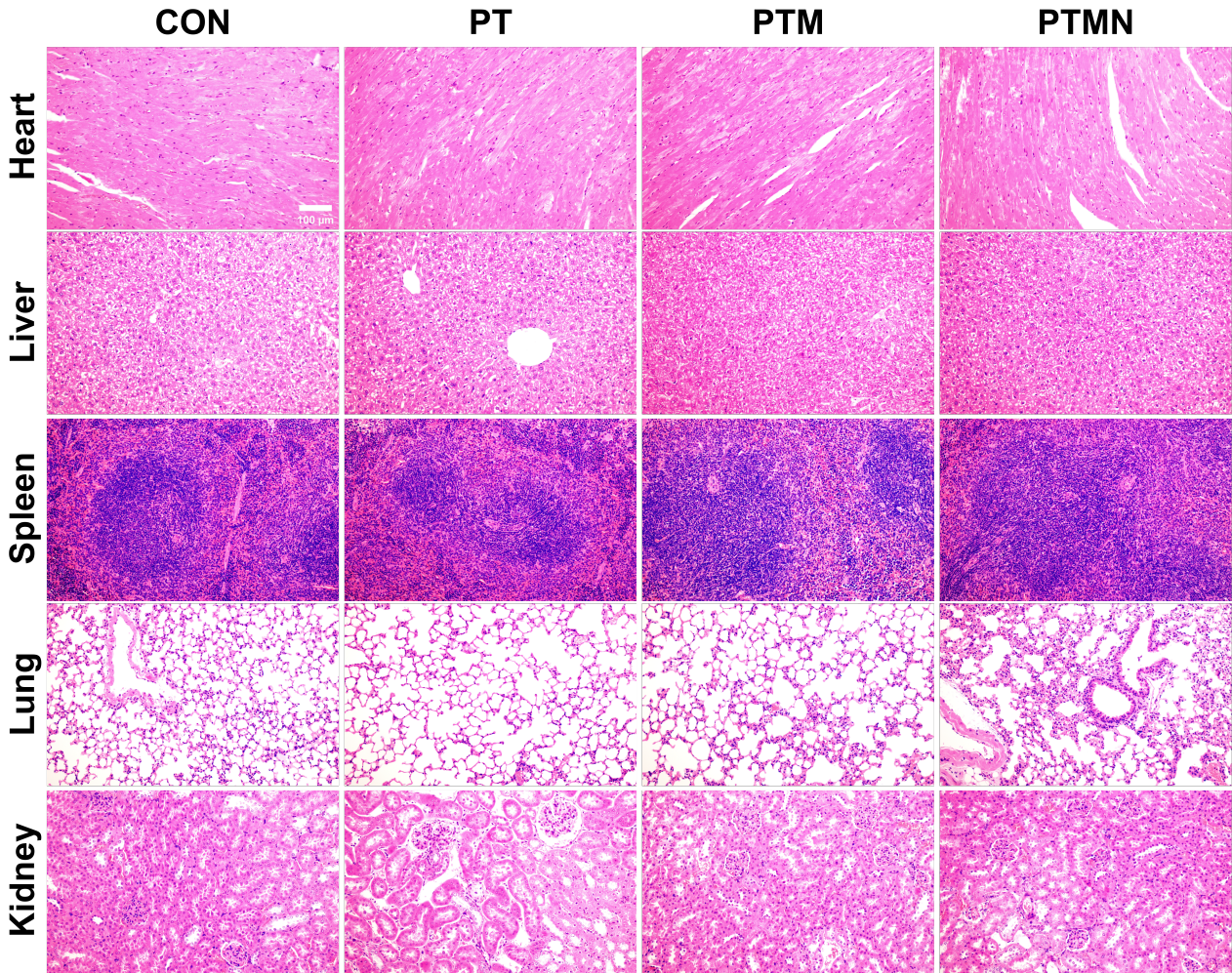


**Figure S13.** HE staining images of major organs in various groups. (scale bar=100um)


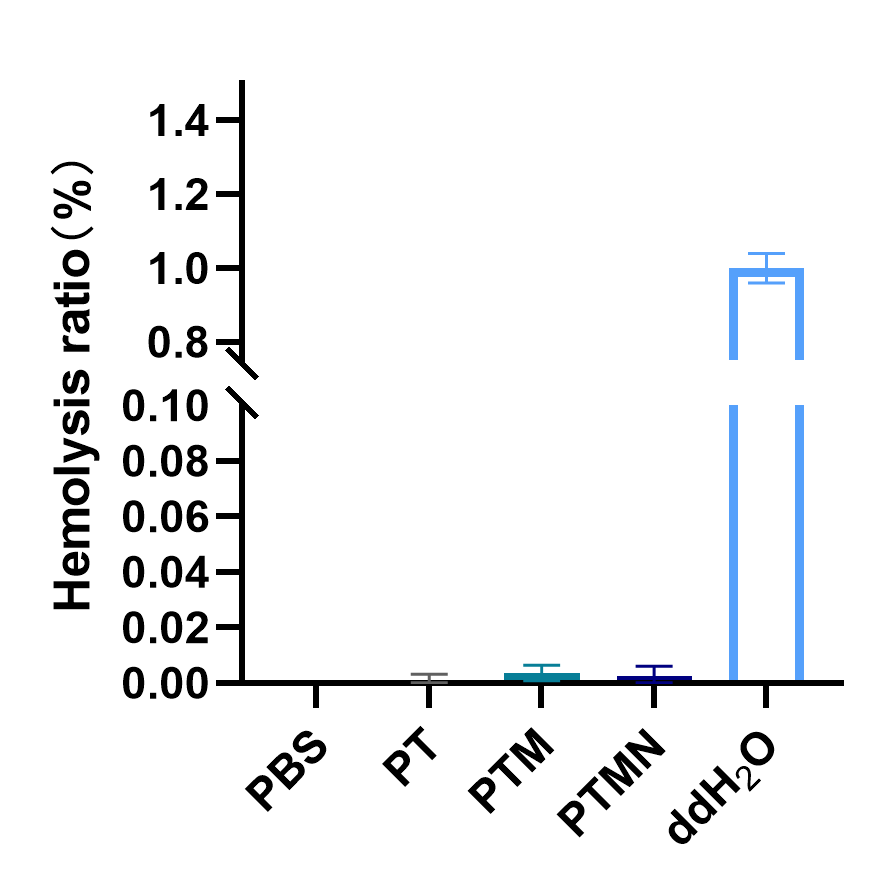


**Figure S14.** Quantitative analysis of hemolysis test (error bars indicate means ± standard deviations).


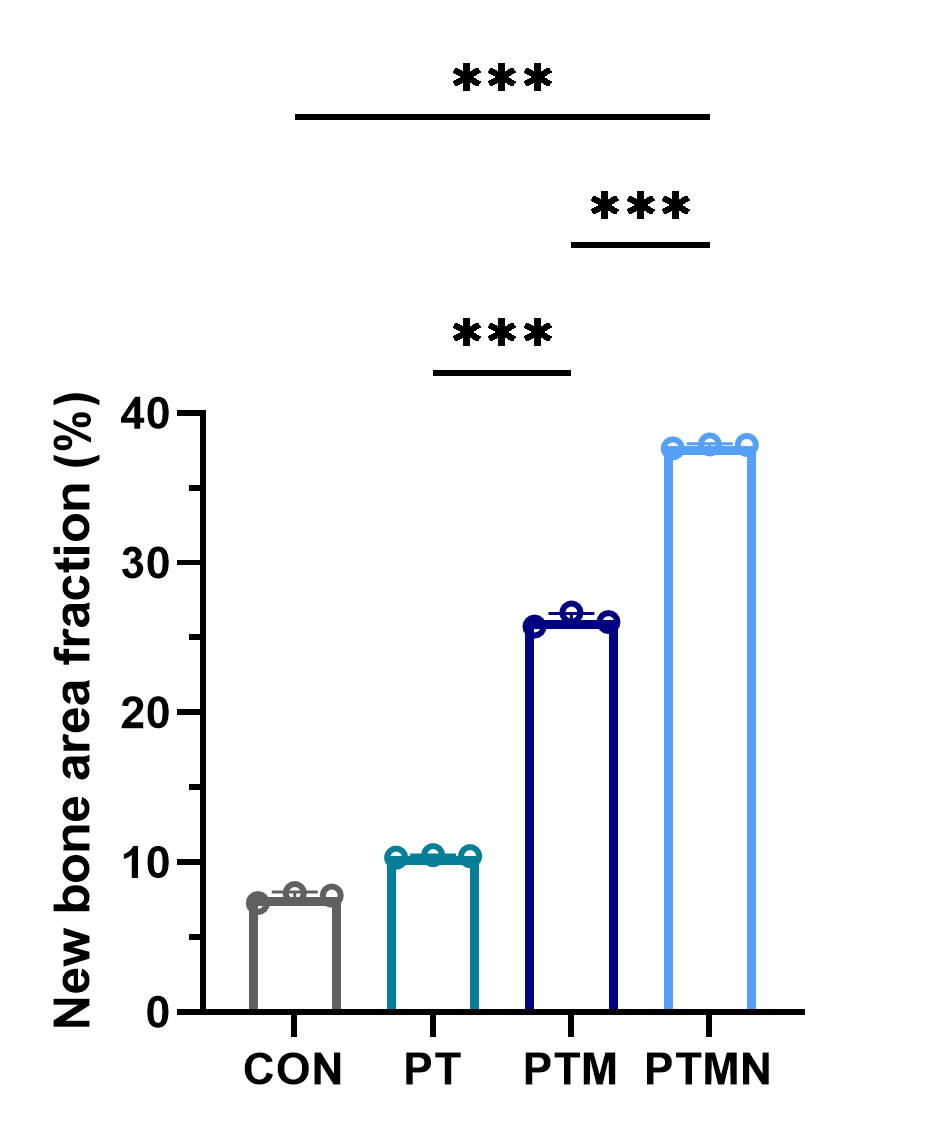


**Figure S15.** Histomorphometrical analysis of new bone area fractions（4W） (**p* < 0.05, ***p* < 0.01, and ****p* < 0.001; “ns” denoted no significant，error bars indicate means ± standard deviations）


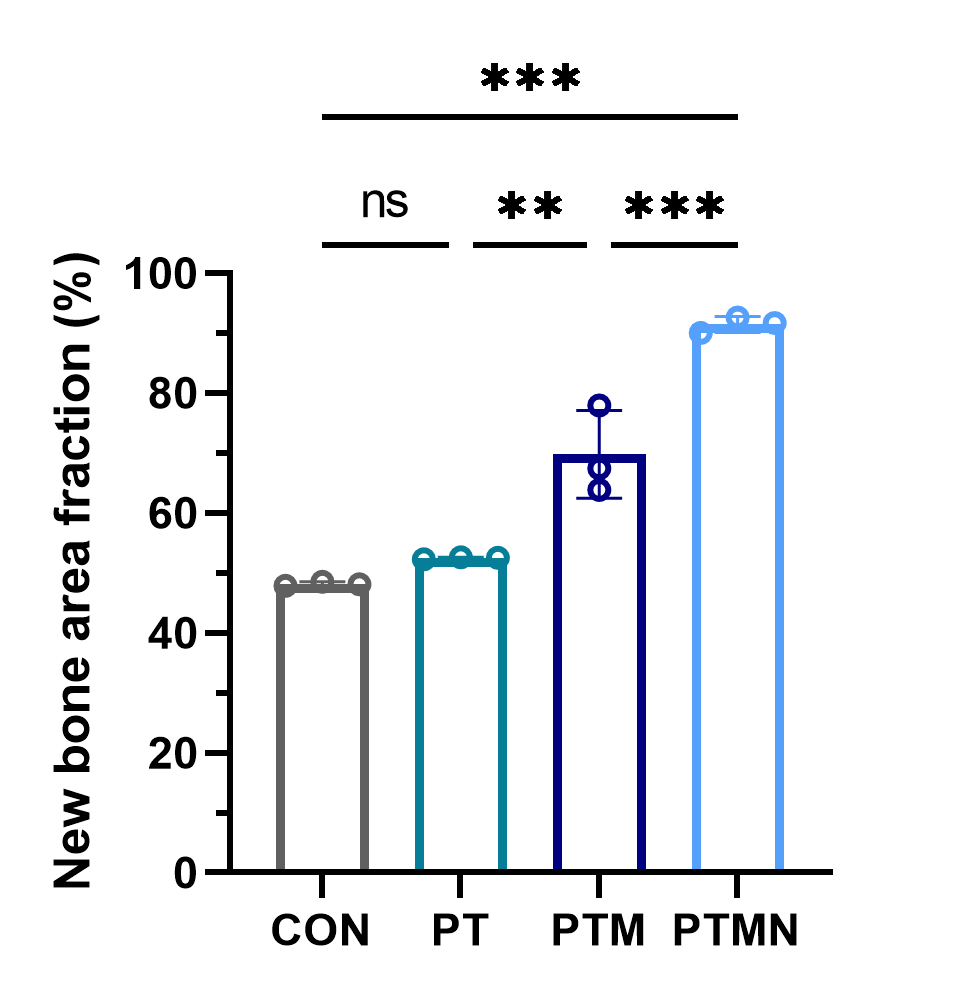


**Figure S16.** Histomorphometrical analysis of new bone area fractions（8W） (**p* < 0.05, ***p* < 0.01, and ****p* < 0.001; “ns” denoted no significant，error bars indicate means ± standard deviations）

1. Xia, H., et al., *Osteogenic Property Regulation of Stem Cells by a Hydroxyapatite 3D-Hybrid Scaffold With Cancellous Bone Structure.* Frontiers in Chemistry, 2021. **9**.

2. Zhang, J., et al., *The size of surface microstructures as an osteogenic factor in calcium phosphate ceramics.* Acta Biomaterialia, 2014. **10**(7): p. 3254-3263.

3. Stiehler, M., et al., *Effect of dynamic 3-D culture on proliferation, distribution, and osteogenic differentiation of human mesenchymal stem cells.* Journal of Biomedical Materials Research Part A, 2009. **89A**(1): p. 96-107.

4. Xie, X.-H., et al., *Biofabrication of a PLGA-TCP-based porous bioactive bone substitute with sustained release of icaritin.* Journal of Tissue Engineering and Regenerative Medicine, 2015. **9**(8): p. 961-972.

5. Negrini, N.C., et al., *An Osteosarcoma Model by 3D Printed Polyurethane Scaffold and In Vitro Generated Bone Extracellular Matrix.* Cancers, 2022. **14**(8).

6. Hernandez, C.J., et al., *The influence of bone volume fraction and ash fraction on bone strength and modulus.* Bone, 2001. **29**(1): p. 74-78.
